# Targeted transcript analysis in muscles from patients with genetically diverse congenital myopathies

**DOI:** 10.1093/braincomms/fcac224

**Published:** 2022-09-02

**Authors:** Christoph Bachmann, Martina Franchini, Luuk R Van den Bersselaar, Nick Kruijt, Nicol C Voermans, Karlijn Bouman, Erik-Jan Kamsteeg, Karl Christian Knop, Lucia Ruggiero, Lucio Santoro, Yoram Nevo, Jo Wilmshurst, John Vissing, Michael Sinnreich, Daniele Zorzato, Francesco Muntoni, Heinz Jungbluth, Francesco Zorzato, Susan Treves

**Affiliations:** Department of Biomedicine, Basel University Hospital, Hebelstrasse 20, Basel 4031, Switzerland; Department of Neurology, Basel University Hospital, Hebelstrasse 20, Basel 4031, Switzerland; Department of Biomedicine, Basel University Hospital, Hebelstrasse 20, Basel 4031, Switzerland; Department of Neurology, Basel University Hospital, Hebelstrasse 20, Basel 4031, Switzerland; Department of Anesthesiology, Malignant Hyperthermia Investigation Unit, Canisius Wilhelmina Hospital, Nijmegen, The Netherlands; Department of Neurology, Donders Institute for Brain, Cognition and Behaviour, Radboud University Medical Center, Nijmegen, The Netherlands; Department of Neurology, Donders Institute for Brain, Cognition and Behaviour, Radboud University Medical Center, Nijmegen, The Netherlands; Department of Neurology, Donders Institute for Brain, Cognition and Behaviour, Radboud University Medical Center, Nijmegen, The Netherlands; Department of Neurology, Donders Institute for Brain, Cognition and Behaviour, Radboud University Medical Center, Nijmegen, The Netherlands; Department of Pediatric Neurology, Donders Institute for Brain, Cognition and Behaviour, Amalia Children’s Hospital, Radboud University Medical Center, Nijmegen, The Netherlands; Department of Clinical Genetics, Radboud Institute for Molecular Life Sciences, Radboud University, Nijmegen Medical Centre, Nijmegen, The Netherlands; Muskelhistologisches Labor, Neurologische Abteilung, Asklepios Klinik St. Georg, Lohmuehlenstraße 5, Hamburg 20099, Germany; Dipartimento di Neuroscienze, Scienze Riproduttive ed Odontostomatologiche, Università degli Studi di Napoli Federico II, Via Pansini 5, Napoli 80131, Italy; Dipartimento di Neuroscienze, Scienze Riproduttive ed Odontostomatologiche, Università degli Studi di Napoli Federico II, Via Pansini 5, Napoli 80131, Italy; Institute of Neurology, Schneider Children’s Medical Center of Israel, Petah Tiqva, Israel; Paediatric Neurology, Red Cross War Memorial Children’s Hospital, Neuroscience Institute, University of Cape Town, Cape Town, South Africa; Department of Neurology, section 8077, Rigshospitalet, University of Copenhagen, Blegdamsvej 9, Copenhagen DK-2100, Denmark; Department of Biomedicine, Basel University Hospital, Hebelstrasse 20, Basel 4031, Switzerland; Department of Neurology, Basel University Hospital, Hebelstrasse 20, Basel 4031, Switzerland; GKT School of Medical Education, King’s College London, Hodgkin Building, Newcomen Street, London SE1 1UL, UK; Dubowitz Neuromuscular Centre and MRC Centre for Neuromuscular Diseases, UCL, Institute of Child Health, London, UK; NIHR Great Ormond Street Hospital Biomedical Research Centre, London, UK; Department of Paediatric Neurology, Neuromuscular Service, Evelina Children’s Hospital, St. Thomas’ Hospital, London, UK; Department of Basic and Clinical Neuroscience, Institute of Psychiatry, Psychology and Neuroscience (IoPPN), King’s College London, London, UK; Randall Center for Cell and Molecular Biophysics, Muscle Signalling Section, Faculty of Life Sciences and Medicine, King’s College, London, UK; Department of Biomedicine, Basel University Hospital, Hebelstrasse 20, Basel 4031, Switzerland; Department of Neurology, Basel University Hospital, Hebelstrasse 20, Basel 4031, Switzerland; Department of Life Science and Biotechnology, University of Ferrara, Via Borsari 46, Ferrara 44100, Italy; Department of Biomedicine, Basel University Hospital, Hebelstrasse 20, Basel 4031, Switzerland; Department of Neurology, Basel University Hospital, Hebelstrasse 20, Basel 4031, Switzerland; Department of Life Science and Biotechnology, University of Ferrara, Via Borsari 46, Ferrara 44100, Italy

**Keywords:** congenital myopathies, mutations, muscle biopsies, transcripts miRNAs, expression

## Abstract

Congenital myopathies are a group of early onset muscle diseases of variable severity often with characteristic muscle biopsy findings and involvement of specific muscle types. The clinical diagnosis of patients typically relies on histopathological findings and is confirmed by genetic analysis. The most commonly mutated genes encode proteins involved in skeletal muscle excitation–contraction coupling, calcium regulation, sarcomeric proteins and thin–thick filament interaction. However, mutations in genes encoding proteins involved in other physiological functions (for example mutations in *SELENON* and *MTM1*, which encode for ubiquitously expressed proteins of low tissue specificity) have also been identified. This intriguing observation indicates that the presence of a genetic mutation impacts the expression of other genes whose product is important for skeletal muscle function. The aim of the present investigation was to verify if there are common changes in transcript and microRNA expression in muscles from patients with genetically heterogeneous congenital myopathies, focusing on genes encoding proteins involved in excitation–contraction coupling and calcium homeostasis, sarcomeric proteins, transcription factors and epigenetic enzymes. Our results identify *RYR1*, *ATPB2B* and miRNA-22 as common transcripts whose expression is decreased in muscles from congenital myopathy patients. The resulting protein deficiency may contribute to the muscle weakness observed in these patients. This study also provides information regarding potential biomarkers for monitoring disease progression and response to pharmacological treatments in patients with congenital myopathies.

## Teaser

In this study, Bachmann *et al*. investigate changes in transcript and microRNA expression in muscles from patients with genetically heterogeneous congenital myopathies (CMs). Their results identify *RYR1*, *ATPB2B* and miRNA-22 as common transcripts whose expression is decreased in muscles from patients harbouring *RYR1*, *SELENON*, *MTM1* and *KBTBD13-*related CMs

## Graphical Abstract

**Graphical abstract:**
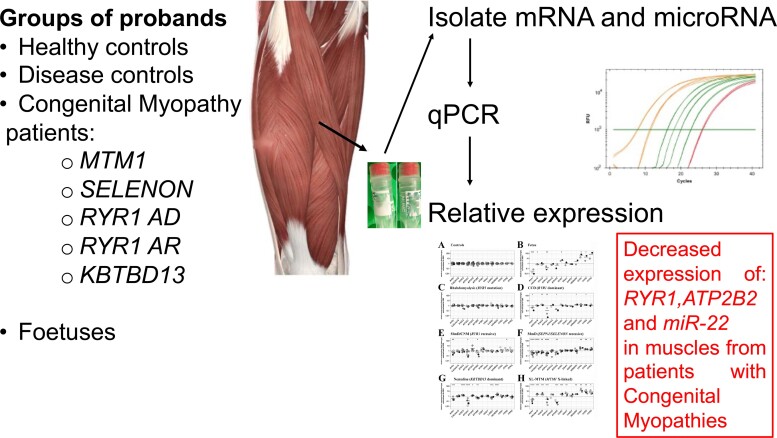

## Introduction

Congenital myopathies (CMs) are a group of generally non-progressive or slowly progressive early onset muscle disorders characterized by muscle weakness and reduced muscle tone. They preferentially affect proximal limb and axial muscles but, depending on the genetic mutation, CMs can impact the function of cardiac, facial, extraocular and respiratory muscles.^1^ Some patients, especially those affected by the more severe forms, may have feeding difficulties and skeletal abnormalities including scoliosis and foot deformities. The overall incidence of CMs as a group is estimated at 1:26 000–1:50 000. Mutations in >20 genes have been reported to date, the most commonly affected genes being those encoding proteins involved in skeletal muscle excitation–contraction coupling (ECC) and calcium homeostasis, thin–thick filament assembly and titin, though mutations in genes encoding ubiquitously expressed proteins are also common.^1–4^ A striking aspect of CMs is their pleiotropy (different clinical and histopathological phenotypes caused by different mutations in one gene) and their genetic heterogeneity (one phenotype caused by mutations in different genes).

Mutations in *RYR1* provide an excellent example of pleiotropy. This gene encodes the ryanodine receptor 1 calcium-release channel (RyR1) of the sarcoplasmic reticulum and mutations therein have been identified in ∼30% of all CM patients.^2,5^ Interestingly, mutations in *RYR1* are linked to various disease phenotypes, including those with dominantly inherited central core disease (CCD, MIM#11700) and mainly recessively inherited subgroups of multiminicore disease (MmD, MIM#255320), centronuclear myopathy (CNM) and congenital fibre type disproportion. Additionally, *RYR1* mutations are the underlying cause of the majority of cases of malignant hyperthermia (MH) susceptibility (MHS, MIM #145600) a pharmacogenetic disorder triggered by depolarizing muscle relaxants or volatile anaesthetics in genetically predisposed individuals. MH-associated *RYR1* mutations have also been implicated in some forms of rhabdomyolysis/exercise induced/heat intolerance,^1,6,7^ King Denborough syndrome a myopathic syndrome characterized by skeletal abnormalities, dysmorphic features and MH susceptibility,^8^ as well as some forms of periodic paralysis.^9^ These results highlight the fact that patients with mutations in one gene may present with a variety of different phenotypes.

MmD, CNM and nemaline myopathy illustrate the genetic heterogeneity of CMs. Indeed, some forms of MmD and particularly the rigid spine muscular dystrophy subtype are caused by recessive mutations in *SELENON* (formerly known as *SEPN1*; MIM#606210). *SELENON* encodes for a ubiquitous selenocysteine-containing protein expressed in the ER of several tissues including brain, lung, spleen and skeletal muscle.^10,11^ CNM can be caused by mutations in a number of genes including *RYR1* (autosomal recessive, AR), *TTN* (AR), *DNM2* (autosomal dominant, AD), *BIN1* (AD and AR), *SPEG* (AR) and *MTM1* (X-linked).^12,13^ The X-linked form (MIM#310400) also known as XL-MTM is by far the most severe subtype of CNMs and is caused by mutations in the lipid phosphatase myotubularin 1 a protein that is expressed in many tissues including brain, liver, endocrine tissues, gastrointestinal tract, kidney and skeletal muscle. Myotubularin de-phosphorylates phosphatidylinositol 3 monophosphate and phosphatidylinositol 3,5-bisphosphate, lipids playing a role in intracellular vesicle trafficking and autophagy.^14^ Interestingly, *MTM1*-related CNM is accompanied by an up-regulation of *DNM2* and this negatively affects the structure and function of triads, suggesting that regulating *DNM2* expression may be a potential target for therapeutic intervention.^15,16^

Finally, nemaline myopathy is linked to mutations in several genes including *NEB*, *ACTA1*, *TPM3*, *KLHL40*, *KLHL41*, *LMOD3*, *KBTBD13*, *MYPN* and more. Many of these genes are implicated in sarcomeric assembly and maintenance and are associated with variable degrees of muscle weakness.^17^ The *KBTBD13*-dominant NEM6 form (MIM#609273) is characterized by progressive proximal and neck weakness, gait abnormalities and exercise intolerance. *KBTBD13* was recently reported to be an actin-binding protein and mutations therein were shown to impair muscle relaxation.^18^

In the present study, we investigated whether the expression of different groups of transcripts are similarly impacted in muscles from CM patients with AD and AR *RYR1*-related myopathies, *SELENON*-related MmD, *KBTBD13*-related nemaline myopathy and *MTM1*-related XL-MTM. The underlying hypothesis being that the primary genetic defects may cause downstream changes in the expression of genes important for muscle function, leading to common biochemical or functional changes. In other words, muscles from patients with different CMs may show similar changes in the expression of transcripts, leading to partially overlapping phenotypes.

In this study, we focus on the expression of transcripts involved in muscle function, ECC, calcium homeostasis, contractile and sarcomeric proteins were impacted. This selection is based on the fact that proteins involved in ECC, calcium regulation and contraction are essential for skeletal muscle contraction and changes in their expression may explain the diminished muscle function. Additionally, changes in the expression of epigenetic enzymes, of transcription factors and of microRNAs (miRNAs), may be causally linked to the altered gene expression which has been observed in some CM.^19–23^

The results show that the expression levels of *RYR1*, *ATPB2B* and miRNA-22 are decreased in muscles from all CM groups. In addition, distinct CMs are characterized by specific transcriptional alterations. The results of this study provide a profile of the changes occurring in muscles from patients affected by different CMs and may be useful in the future to help classify patients, monitor disease progression and potentially evaluate the response to pharmacological treatment.

## Materials and methods

### Compliance with ethical standards

All procedures were performed in accordance with the ethical standards of the institutional and/or national research committee and with the 1964 Helsinki declaration and its later amendments or comparable ethical standards. This study was approved by the Ethikkommission Nordwest- und Zentralschweiz (permit N° EKNZ 2014-065); all subjects gave written informed consent to carry out this work.

### Muscle biopsies

Quadriceps muscle biopsies from patients with genetically confirmed variants and healthy non-affected individuals undergoing the *in vitro* contracture test were used. For some patients, residual material of needle biopsies for other diagnostic purposes was used. The patients with the rhabdomyolysis/exercise intolerance phenotype had previously presented elevated creatine kinase (CK) levels (CK > 10 000 IU/l). All healthy controls underwent *RYR1* sequencing and none were found to carry any *RYR1* variants.

### Quantitative polymerase chain reaction

Was performed as previously described.^19^ Briefly, RNA was isolated from muscle biopsies using TRIzol™ reagent (Thermo Fischer; 15596026) following the manufacturer’s protocol. For subsequent quantitative real-time polymerase chain reaction (qPCR), 1000 ng of RNA were reverse-transcribed to cDNA using the high-capacity cDNA reverse transcription kit (Applied Biosystems; 4368814) on an Applied Biosystems 2720 Thermal Cycler. The cDNA was amplified using PowerUP™ Sybr™ Green Master Mix (Applied Biosystems; A25742) for regular DNA quantification on an Applied Biosystems 7500 Fast Real-time PCR System running 7500 software version 2.3. A complete list of the transcripts whose expression was investigated is presented in Supplementary Table 1.

Each reaction was performed in duplicate and results are expressed as log_2_-fold change relative to controls; gene expression was normalized to the muscle-specific housekeeping gene *DESMIN* (*DES*). *DES* was selected as a housekeeping gene, since desmin is expressed at high levels in all vertebrate muscle cells, as opposed to *GAPDH* which is expressed in all mammalian cells. A table comparing the *C_t_* values for *DES* and *GAPDH* in muscles from controls and patients with AD-*RYR1* mutations and *SEPN1* mutations is shown in Supplementary Table 2. The sequences of the primers used are listed in Supplementary Table 3.

Because of the large number of samples, each 96-well PCR plate contained only samples from healthy controls and from a specific disease group (or foetal muscles). Furthermore, since the amount of biological material was very limited, for some patients the expression levels of a restricted number of transcripts were evaluated. Data were centred using average Δ*C_t_* value of the controls which were included in each 96-well PCR plate. Results are presented as log_2_-fold change in patients/foetal muscles relative to healthy controls.

### MiRNA quantification

For quantification of miRNAs, 450 ng of RNA isolated from biopsies using TRIzol™ were treated with TaqMan™ miRNA reverse transcription kit (Applied Biosystems; 4366596) on an Applied Biosystems 2720 Thermal Cycler using a miRNA primer mix, as previously described.^19^ Subsequent qPCR was performed using TaqMan™ Universal Master Mix II, no UNG (Applied Biosystems; 4440040) according to the manufacturer’s protocol using specific miRNA probes listed in Supplementary Table 4. Quantification was performed on an Applied Biosystems 7500 Fast Real-time PCR System running 7500 software version 2.3 and each reaction was performed in duplicate. Results were normalized to the human housekeeping snRNA U6. Each 96-well PCR plate contained only samples from healthy controls and from a specific disease group (or foetal muscles). Data were centred using average Δ*C_t_* value of the controls included in each 96-well PCR plate. Results are presented as log_2_-fold change in patients/foetal muscles, relative to healthy controls.

### Statistical analysis

Since each test plate contained only samples from the control group and a specific disease group (or foetuses), statistical analysis was only made between these two groups. Statistical analysis was performed using ‘R’ version 4.2.0 running on platform x86_64-apple-darwin13.4.0 (64 bits). Log_2_-fold changes and standard errors were estimated using the linear model fitted to every gene, with disease as an explanatory variable. Moderated *t*-statistics was calculated using the limma package.^24^ Obtained *P*-values were adjusted for multiple testing using Benjamini–Hochberg method to control the false discovery rate.^25^ All quantitative PCR and miRNA figures were created using R Studio (version 1.4.1106) and assembled to panels using Adobe Photoshop CS6.

### Data availability

Data sharing is not applicable, since all results are included in the manuscript.

## Results

### Selection of patients and mutations

Previous investigations have demonstrated that muscles from patients with CMs carrying mutations in *RYR1*, *SELENON* and *MTM1* have reduced levels of RyR1.^19–23^ In the present investigation, we expanded the study and directly compared the expression of transcripts encoding other proteins involved in ECC and calcium homeostasis, myofilaments, transcription factors and regulators, epigenetic enzymes (see Supplementary Table 1 for the transcripts that were analysed and summary of the function of the encoded proteins) as well as selected families of miRNAs, in muscle biopsies from patients who had previously undergone diagnostic genetic testing. We also included disease controls, namely samples from patients carrying dominant *RYR1* mutations associated with exertional rhabdomyolysis/exercise intolerance and foetal muscles. Supplementary Table 5 shows the phenotypes and genotypes of the patients we investigated. A total of 50 patients were included and pathogenic mutations were identified in 96% (48/50) of them. In particular, 13 probands were diagnosed as having rhabdomyolysis/exercise intolerance having suffered one or more episodes of exertional rhabdomyolysis (CK > 10 000 IU/l). All carried *RYR1* mutations and were also classified as MHS by the *in vitro* contracture test. Three probands were diagnosed as having AD CCD of which two carried pathogenic *RYR1* mutations. Eight probands were diagnosed as having AR MmD/CNM and carried *RYR1* mutations (in one patient, the second mutation was not identified). Fourteen probands were diagnosed as having AR MmD and carried *SELENON* mutations. Six probands originating from two families were diagnosed as having AD NEM6 (*KBTBD13* nemaline myopathy). Six probands were diagnosed as having XL-MTM and carried *MTM1* mutations. Classification of the variants identified in the patients and reference sequence of the mutated genes are presented in Supplementary Table 6. Muscle biopsies from 12 healthy non-affected individuals were used as control. Transcript expression levels in muscles of patients/foetal muscles were compared with those observed in muscles from healthy controls, which were set to 1. Gene expression could not be evaluated for all genes in all probands because of the lack of a sufficient amount of biological material.

### Expression of transcripts encoding proteins involved in ECC and calcium homeostasis

**Figure 1:**
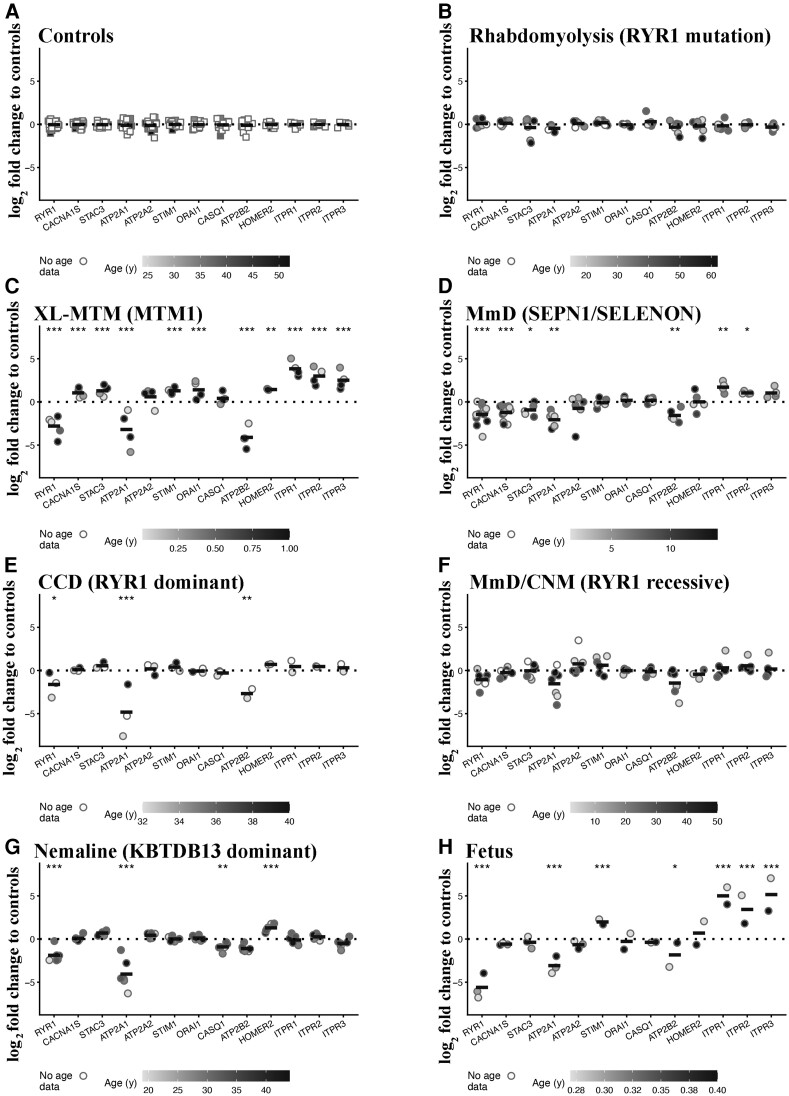
**Muscles from patients with CM show significant changes in the expression levels of transcripts encoding proteins involved in ECC and calcium homeostasis.** Expression levels of the indicated transcripts were determined by qPCR and normalized to the expression of *DES*. Muscle biopsies were from: (**A**) healthy controls; (**B**) patients with exertional rhabdomyolysis/heat stroke/exercise intolerance carrying *RYR1* mutations; (**C**) patients with *MTM1-*related XL-MTM; (**D**) patients with AR *SELENON*-related MmD; (**E**) patients with AD-*RYR1*-related CCD; (**F**) patients with AR *RYR1*-related MmD/CNM; (**G**) patients with AD *KBTBD13-*related nemaline myopathy; (**H**) foetuses. The greyscale given to the symbols reflects the age range of the patients and the scale bar at the bottom of each panel correlates greyscale to age. Empty symbols represent patients or probands whose age was not known. Square symbols represent results from controls; circles represent results from disease patients and foetuses. The relative transcript expression in patient muscles was compared with that in muscles from healthy controls that was set to 1. Statistical analysis was performed using the ‘R’ version 4.2.0 running on platform x86_64-apple-darwin13.4.0 (64 bits). Comparisons of each disease group (or foetus) to controls were calculated using the limma package^24^ of ‘R’. Obtained *P*-values were adjusted for multiple testing using Benjamini–Hochberg method to control the false discovery rate. Means were considered statistically significant, when the adjusted *P*-values were <0.05. The horizontal black bar represents the mean content levels in patient muscles. *P < 0.05; **P < 0.01; ***P < 0.001.

Figure 1 shows the results obtained when analysing transcripts encoding the main proteins involved in skeletal muscle ECC and calcium homeostasis in each group of patients with the different panels divided by disease and genotype. Each symbol represents the average value (of duplicate runs) obtained from a single patient plotted on a log_2_ scale with the horizontal line representing the mean value.

None of the transcripts examined encoding proteins involved in ECC and calcium homeostasis^26–35^ varied between muscles from healthy controls (Fig. 1A) and disease controls, i.e. patients with *RYR1*-related rhabdomyolysis/exercise intolerance (Fig. 1B). Muscles from patients with *MTM1* mutations showed the greatest changes in transcript levels, with all transcripts except *ATP2A2* and *CASQ1*, being significantly altered (Fig. 1C). In particular, *RYR1* transcript levels were reduced by almost 10-fold (the mean log_2_-fold change was −2.75 adjusted *P* = 2.89E − 09), whereas the transcript levels encoding the three InsP3R isoforms were significantly increased (the mean log_2_-fold changes for *ITPR1*, *ITPR2* and *ITPR3* were 3.86 adjusted *P* = 1.35E − 09, 3.00 adjusted *P* = 1.18E − 08 and 2.53 adjusted *P* = 1.62E − 04, respectively). The expression levels of *CACNA1S*, *STAC3*, *STIM1*, *ORAI1* and *HOMER* were significantly increased (the mean log_2_-fold changes were 1.06 adjusted *P* = 2.22E − 04, 1.32 adjusted *P* = 4.33E − 04, 1.30 adjusted *P* = 3.07E − 05, 1.44 adjusted *P* = 8.82E − 06 and 1.46 adjusted *P* = 0.001, respectively). Muscles from patients with AR *SELENON* MmD also showed significantly altered expression levels of several transcripts (Fig. 1D), including lower levels of transcripts encoding *RYR1*, *CACNA1S*, *STAC3*, *ATP2A1* and *ATP2B2* (the mean log_2_-fold changes were −1.39 adjusted *P* = 6.61E − 05, −1.20 adjusted *P* = 2.35E − 08, −0.89 adjusted *P* = 0.016, −1.95 adjusted *P* = 0.0034 and −1.45 adjusted *P* = 0.0077, respectively) and increased levels of *ITPR1* and *ITPR2* (the mean log_2_-fold changes were 1.73 adjusted *P* = 0.0013 and 1.05 adjusted *P* = 0.021, respectively).

Importantly, in muscles from all other CM patients the levels of *RYR1* and *ATP2B2* were reduced compared with healthy controls though they did not always reach significance when the adjusted *P*-values were used. Their mean log_2_-fold change levels in patients with AD-*RYR1*-related CCD were −1.58 (adjusted *P* = 0.081) and −2.56 (adjusted *P* = 0.0025; Fig. 1E), in patients with AR *RYR1*-related MmD/CNM, they were reduced to −1.03 (adjusted *P* = 0.063) and −1.34 (adjusted *P* = 0.052; Fig. 1F), and in AD nemaline myopathy patients, they were −1.85 (adjusted *P* = 8.74E − 05) and −0.98 adjusted (*P* = 0.076; Fig. 1G). Foetal muscles have a distinct transcript expression pattern (Fig. 1H), showing a more than 10-fold reduction of *RYR1* (the mean log_2_-fold change was −5.56 adjusted *P* = 1.10E − 14) and *ATP2A1* (the mean log_2_-fold change was −2.97 adjusted *P* = −8.41E − 04) and significantly higher levels of *STIM1*, *ITPR1*, *ITPR2* and *ITPR3* (mean log_2_-fold changes were 1.99 adjusted *P* = 4.34E − 06, 5.02 adjusted *P* = 1.67E − 09, 3.44 adjusted *P* = 1.61E − 07 and 5.17 adjusted *P* = 1.45E − 07, respectively; see Supplementary Table 7 for complete data set and statistical analysis).

In conclusion, these results show that there are specific changes in the expression levels of several transcripts in muscles from all patients with CM, in particular: (i) decreased levels of *RYR1* and *ATP2B2*; (ii) increased levels of transcripts encoding for one or more of *ITPR* isoforms; and (iii) diseases which more strongly impact muscle function such as XL-MTM1 were accompanied by the greatest changes in the expression of transcripts encoding proteins involved in calcium homeostasis. Importantly, muscles from patients carrying dominant *RYR1* mutations linked to rhabdomyolysis did not exhibit any changes in the expression of the investigated transcripts.

### Expression of transcripts encoding contractile and sarcomeric proteins

**Figure 2:**
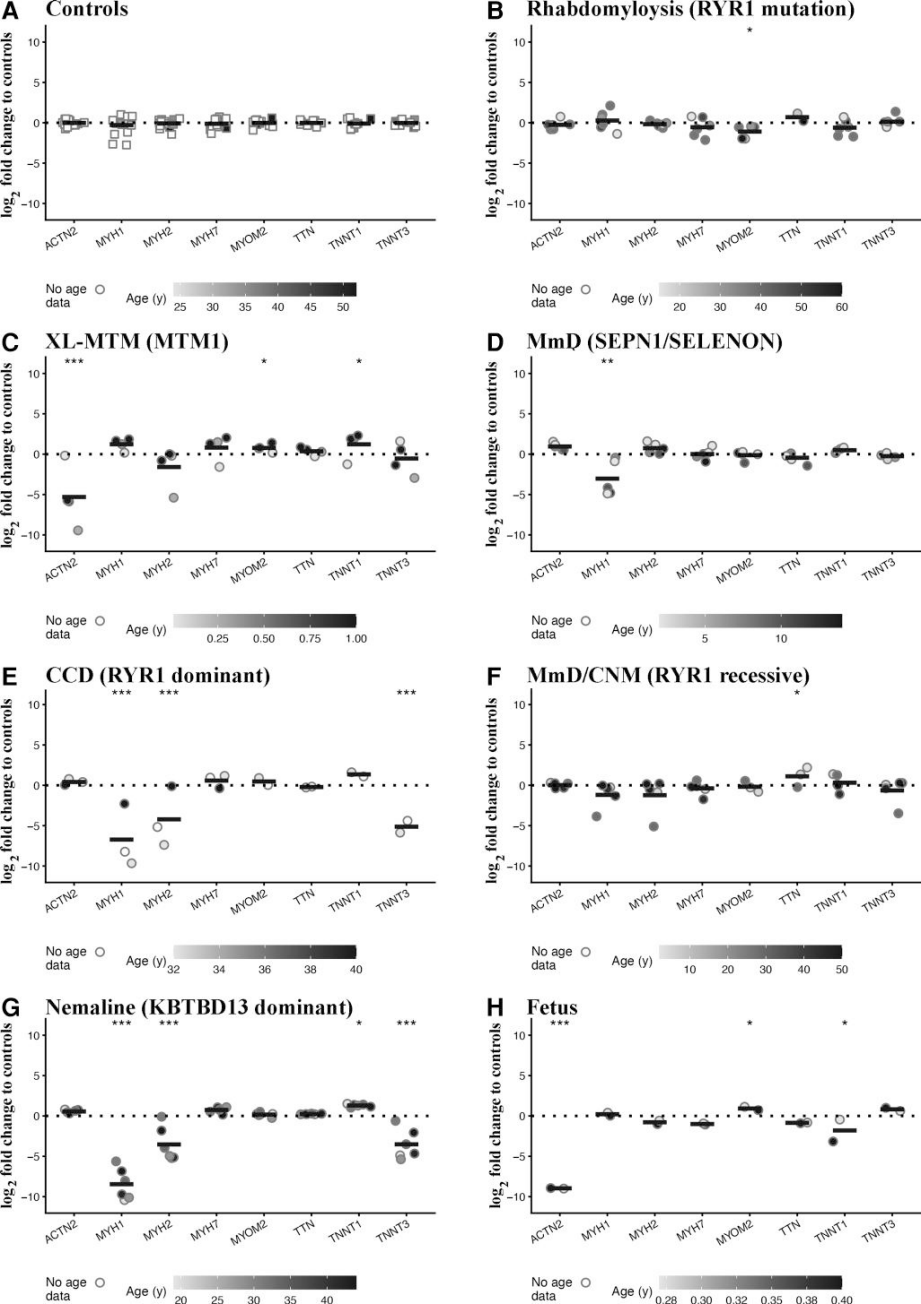
**Muscles from patients with rhabdomyolysis and congenital myopathies show significant changes in the expression levels of transcripts encoding contractile and sarcomeric proteins.** Expression levels of the indicated transcripts were determined by qPCR and normalized to the expression of *DES*. Muscle biopsies were from: (**A**) healthy controls; (**B**) patients with exertional rhabdomyolysis/heat stroke/exercise intolerance carrying *RYR1* mutations; (**C**) patients with *MTM1-*related XL-MTM; (**D**) patients with AR *SELENON*-related MmD; (**E**) patients with AD-*RYR1*-related CCD; (**F**) patients with AR *RYR1*-related MmD/CNM; (**G**) patients with AD *KBTBD13-*related nemaline myopathy; (**H**) foetuses. The greyscale given to the symbols reflects the age range of the patients and the scale bar at the bottom of each panel correlates greyscale to age. Empty symbols represent patients or probands whose age was not known. Square symbols represent results from controls; circles represent results from disease patients and foetuses. The relative transcript expression in patient muscles was compared with that in muscles from healthy controls that was set to 1. Statistical analysis was performed using ‘R’ version 4.2.0 running on platform x86_64-apple-darwin13.4.0 (64 bits). Comparisons of each disease group (or foetus) to controls were calculated using the limma package^24^ of ‘R’. Obtained *P*-values were adjusted for multiple testing using Benjamini–Hochberg method to control the false discovery rate. Means were considered statistically significant when the adjusted *P*-values were <0.05. The horizontal black bar represents the mean content levels in patient muscles. *P < 0.05; **P < 0.01; ***P < 0.001.

Muscle groups specialized for different tasks express different myofilament isoforms whose shortening velocity is determined by the expression of specific myosin heavy chain (MyHC) isoforms.^36^ Additionally, there are many proteins involved in the assembly and organization of the contractile apparatus with mutations in genes encoding some sarcomeric proteins are linked to specific forms of CMs.^1^ In this part of the study, we investigated changes in the expression of a subset of these transcripts, focusing on the transcripts encoding MyHC isoforms, α-actinin, myomesin, titin and troponin T.^36,37^ Interestingly, muscle biopsies from disease controls, i.e. patients with *RYR1*-related rhabdomyolysis/exercise intolerance, showed significantly lower expression levels of *MYOM2* (the mean log_2_-fold change was −1.07 adjusted *P* = 0.01; Fig. 2B) compared with healthy controls. On the other hand, muscles from patients with *MTM1* mutation showed increased expression levels of *MYOM2* and *TNNT1* (the mean log_2_-fold changes were 0.79 adjusted *P* = 0.01 and 1.32 adjusted *P* = 0.01, respectively) and decreased levels of *ACTN2* (the mean log_2_-fold change was −5.26 adjusted *P* = 1.03E − 10; Fig. 2C). Muscles from patients with AR *SELENON-*related MmD showed decreased levels of *MYH1* (the mean log_2_-fold change was −2.74 adjusted *P* = 0.003; Fig. 2D). Compatible with a loss of fast twitch fibres, muscle biopsies from patients with AD-*RYR1*-related CCD showed a significant decrease in *MYH1*, *MYH2* and *TNNT3* (mean log_2_-fold changes were −6.43 adjusted *P* = 9.09E − 07, −4.13 adjusted *P* = 7.29E − 04 and −5.10 adjusted *P* = 2.36E − 05, respectively; Fig. 2E), whereas those from AR *RYR1-*related MmD/CNM showed a significant increased expression of *TTN* (the mean log_2_-fold change was 1.13 adjusted *P* = 0.03; Fig. 2F). Interestingly, muscles from patients with AD *KBTBD13-*related nemaline myopathy who also have a loss of fast twitch fibres, showed reduced expression levels of all *MYH1*, *MYH2* and *TNNT3* (the mean log_2_-fold changes were −8.17 adjusted *P* = 2.78E − 13, −3.44 adjusted *P* = 1.55E − 04 and −3.48 adjusted *P* = 1.55E − 05, respectively) and increased expression of *TNNT1* (the mean log_2_-fold change was 1.39 adjusted *P* = 0.01; Fig. 2G). Foetal muscles (Fig. 2H) showed a >200-fold lower expression level of *ACTN2* (the mean log_2_-fold change was −8.95 adjusted *P* = 7.69E − 14), increased levels of *MYOM2* (the mean log_2_-fold change was 0.944 adjusted *P* = 0.03) and decreased levels of *TNNT1* (the mean log_2_-fold change was −1.70 adjusted *P* = 0.02; see Supplementary Table 8 for the complete data set and statistical analysis).

In short, (i) muscles from patients with *MTM1* mutations showed increased levels of *TNNT1* and, similar to foetal muscles, very low expression levels of *ACTN2*; (ii) muscles from patients with *SELENON*-related MmD showed a decrease of *MYH1* expression; (iii) compatible with the loss of fast twitch Type 2 fibres, muscles from patients with CCD and nemaline myopathy exhibit decreased expression of genes encoding *MYH1*, *MYH2* and *TNNT3* and increased expression levels of *TNNT1*; and (iv) muscles from rhabdomyolysis/exercise intolerance individuals with dominant *RYR1* mutations showed decreased levels of *MYOM2*.

### Expression of transcripts encoding enzymes involved in epigenetic modifications

**Figure 3:**
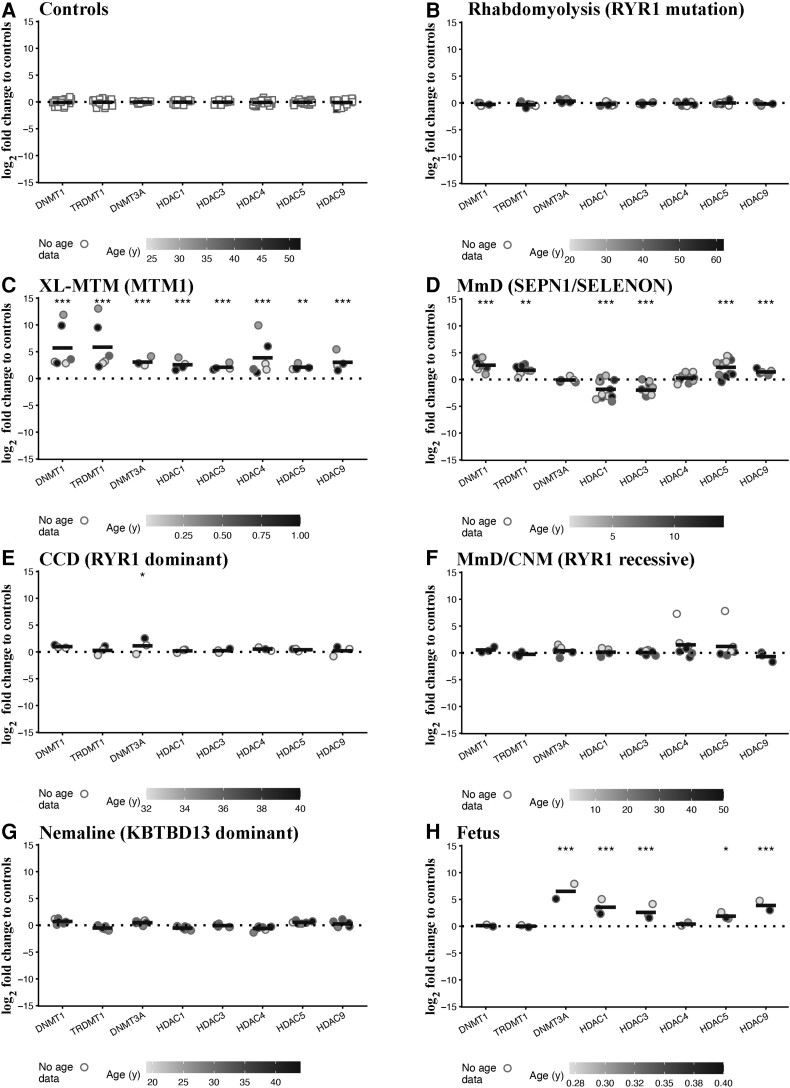
**Muscles from patients with congenital myopathies show significant changes in the expression levels of transcripts encoding enzymes involved in epigenetic modifications.** Expression levels of the indicated transcripts were determined by qPCR and normalized to the expression of *DES*. Muscle biopsies were from: (**A**) healthy controls; (**B**) patients with exertional rhabdomyolysis/heat stroke/exercise intolerance carrying *RYR1* mutations; (**C**) patients with *MTM1-*related XL-MTM; (**D**) patients with AR *SELENON*-related MmD; (**E**) patients with AD *RYR1*-related CCD; (**F**) patients with AR *RYR1*-related MmD/CNM; (**G**) patients with AD *KBTBD13-*related nemaline myopathy; (**H**) foetuses. The greyscale given to the symbols reflects the age range of the patients and the scale bar at the bottom of each panel correlates greyscale to age. Empty symbols represent patients or probands whose age was not known. Square symbols represent results from controls; circles represent results from disease patients and foetuses. The relative transcript expression in patient muscles was compared with that in muscles from healthy controls that was set to 1. Statistical analysis was performed using ‘R’ version 4.2.0 running on platform x86_64-apple-darwin13.4.0 (64 bits). Comparisons of each disease group (or foetus) to controls were calculated using the limma package^24^ of ‘R’. Obtained *P*-values were adjusted for multiple testing using Benjamini–Hochberg method to control the false discovery rate. Means were considered statistically significant when the adjusted *P*-values were <0.05. The horizontal black bar represents the mean content levels in patient muscles. *P < 0.05; **P < 0.01; ***P < 0.001.

We subsequently investigated the levels of transcripts encoding epigenetic enzymes including DNA methyl transferases (DNMTs) and Classes I and II histone deacetylases (HDACs). We hypothesize that the activity of these enzymes may be responsible in part, for the altered expression levels of muscle transcripts since they strongly influence the structure of chromatin, by rendering it more accessible (euchromatin) or less accessible (heterochromatin) to transcription.^38,39^ Our results show that the expression levels of DNMTs and HDACs were similar in muscles from healthy controls (Fig. 3A) and disease controls, i.e. patients with *RYR1*-related exertional rhabdomyolysis (Fig. 3B). On the other hand, muscles from XL-MTM patients showed a significant increase in all *DNMT* and *HDAC* isoforms examined (Fig. 3C). The mean log_2_-fold changes of transcripts encoding *DNMT1*, *TRDMT1* (DNMT2) and *DNMT3A* were 5.79 adjusted (*P* = 6.50E − 11), 5.91 adjusted (*P* = 6.96E − 11) and 3.09 (adjusted *P* = 3.71E − 10), respectively. The levels of Class I HDACs including *HDAC1* and *HDAC3* were significantly increased (the mean log_2_-fold changes were 2.61 adjusted *P* = 4.16E − 06 and 2.14 adjusted *P* = 2.66E − 07, respectively) as were those of Class IIa HDACs, including *HDAC4*, *HDAC5* and *HDAC9* (the mean log_2_-fold changes were 3.95 adjusted *P* = 6.83E − 08, 2.16 adjusted *P* = 0.001 and 3.14 adjusted *P* = 1.18E − 09, respectively). Muscles from patients with AR *SELENON* also showed significantly increased expression levels of *DNMT1* and *TRDMT1* (the mean log_2_-fold changes were 2.75 adjusted *P* = 6.61E − 05 and 1.79 adjusted *P* = 0.006, respectively), of *HDAC5* and *HDAC9* (the mean log_2_-fold changes were 2.33 adjusted *P* = 5.59E − 06 and 1.51 adjusted *P* = 9.92E − 04, respectively) and decreased levels of Class I HDACs (the mean log_2_-fold change of *HDAC1* and *HDAC3*, were −1.78 adjusted *P* = 7.78E − 06 and −1.94 adjusted *P* = 6.83E − 10, respectively; Fig. 3D). In muscles from patients with AD-*RYR1*-related CCD *DNMT3A* expression levels were increased (the mean log_2_-fold change was 1.16 adjusted *P* = 0.03), whereas in AR *RYR1*-related MmD/CNM, *HDAC4* expression levels were increased though they did not achieve statistical significance (the mean log_2_-fold change was 1.58 adjusted *P* = 0.06; Fig. 3F). Interestingly, the levels (fold change) of transcript encoding epigenetic enzymes were not impacted in muscles from patients with AD nemaline myopathy (Fig. 3G) and in AD-*RYR1*-related CCD only, the expression level of *DNMT3A* was increased (the mean log_2_-fold change was 1.16 adjusted *P* = 0.03; Fig. 3E). Foetal muscles showed a significant increase in *DNMT3A* (the mean log_2_-fold change was 6.53 adjusted *P* = 7.28E − 16) as well as in *HDAC1*, *HDAC3*, *HDAC5* and *HDAC9* expression (the mean log_2_-fold changes were 3.58 adjusted *P* = 1.26E − 07, 2.62 adjusted *P* = 8.71E − 08, 1.92 adjusted *P* = 0.01 and 3.98 adjusted *P* = 4.56E − 08, respectively; Fig. 3H, see Supplementary Table 9 for the complete data set and statistical analysis).

Taken together, these results show that muscles of CM patients (excluding *KBTBD13*-related nemaline myopathy patients) as well as foetal muscles exhibit increased levels of transcripts encoding DNMTs and/or mainly, Class II HDACs. Patients carrying *RYR1* mutations linked to exertional rhabdomyolysis/exercise intolerance did not show changes in the expression of any of the investigated transcripts encoding epigenetic enzymes.

### Expression of transcripts encoding skeletal muscle transcription factors and splicing regulators

**Figure 4:**
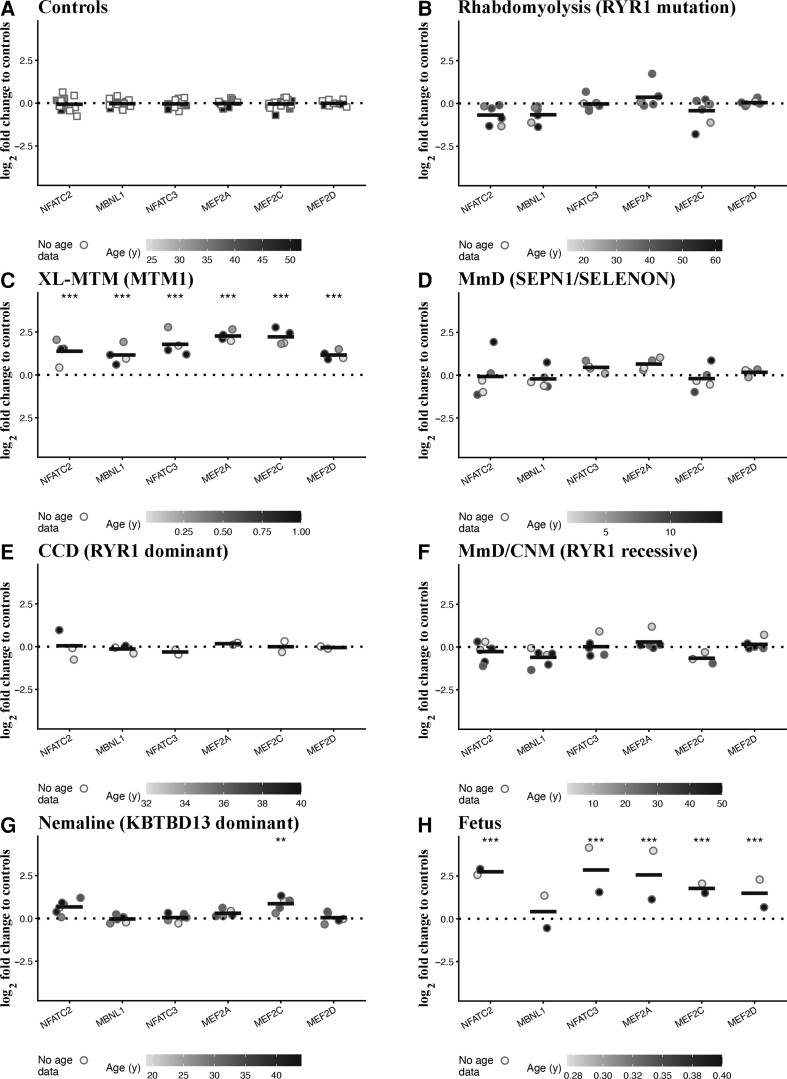
**Muscles from patients with congenital myopathies show significant changes in the expression levels of transcripts encoding transcription factors and splicing regulators.** Expression levels of the indicated transcripts were determined by qPCR and normalized to the expression of *DES*. Muscle biopsies were from: (**A**) healthy controls; (**B**) patients with exertional rhabdomyolysis/heat stroke/exercise intolerance carrying *RYR1* mutations; (**C**) patients with *MTM1-*related XL-MTM; (**D**) patients with AR *SELENON*-related MmD; (**E**) patients with AD *RYR1*-related CCD; (**F**) patients with AR *RYR1*-related MmD/CNM; (**G**) patients with AD *KBTBD13-*related nemaline myopathy; (**H**) foetuses. The greyscale given to the symbols reflects the age range of the patients and the scale bar at the bottom of each panel correlates greyscale to age. Empty symbols represent patients or probands whose age was not known. Square symbols represent results from controls; circles represent results from disease patients and foetuses. The relative transcript expression in patient muscles was compared with that in muscles from healthy controls that was set to 1. Statistical analysis was performed using ‘R’ version 4.2.0 running on platform x86_64-apple-darwin13.4.0 (64 bits). Comparisons of each disease group (or foetus) to controls were calculated using the limma package^24^ of ‘R’. Obtained *P*-values were adjusted for multiple testing using Benjamini–Hochberg method to control the false discovery rate. Means were considered statistically significant when the adjusted *P*-values were <0.05. The horizontal black bar represents the mean content levels in patient muscles. *P < 0.05; **P < 0.01; ***P < 0.001.

We next examined the levels of a subset of skeletal muscle transcription factors, including NFAT and MEF2 family members,^40–42^ and of muscleblind (MBNL1).^43^ The transcript expression levels in muscles from disease controls, i.e. patients with *RYR1*-related rhabdomyolysis/exercise intolerance were similar to those found in muscles from controls (Fig. 4B). Muscles of XL-MTM patients showed the greatest changes in transcript expression levels and importantly, the expression of all investigated transcripts was significantly increased (Fig. 4C). The expression (the mean log_2_-fold changes were: 1.44 adjusted *P* = 2.22E − 04 for *NFATC2*, 1.83 adjusted *P* = 3.48E − 0.6 for *NFATC3*, 2.29 adjusted *P* = 1.17E − 07 for *MEF2A*, 2.26 adjusted *P* = 8.43E − 09 *MEF2C*, 1.17 adjusted *P* = 8.82E − 06 for *MEF2D* and 1.19 adjusted *P* = 4.84E − 05 for *MBNL1*). None of the investigated transcripts were changed in muscles from patients with *SELENON-*related MmD (Fig. 4D) nor from patients with AD-*RYR1*-related CCD (Fig. 4E) though in AR *RYR1* MmD/CNM *MBNL1* levels were reduced though the fold change did not achieve significance (the mean log_2_-fold change was −0.57 adjusted *P* = 0.06; Fig. 4F). *MEF2C* levels were significantly increased in muscles of patients with AD nemaline myopathy (the mean log_2_-fold change was 0.90 adjusted *P* = 0.006; Fig. 4G). Foetal muscles also showed significant increased expression levels of all the transcripts examined except for MBNL1 whose levels were unchanged (Fig. 4H; see Supplementary Table 10 for the complete data set and statistical analysis).

These results indicate that in the muscles from CM patients: (i) *NFATC2* and *NFATC3* levels do not correlate with the expression of specific slow twitch muscle genes and (ii) the expression level of different *MEF2* isoforms increases in muscles from XL-MTM and AD nemaline myopathy patients.

### Expression of miRNAs in muscles from patients with different muscle diseases

**Figure 5:**
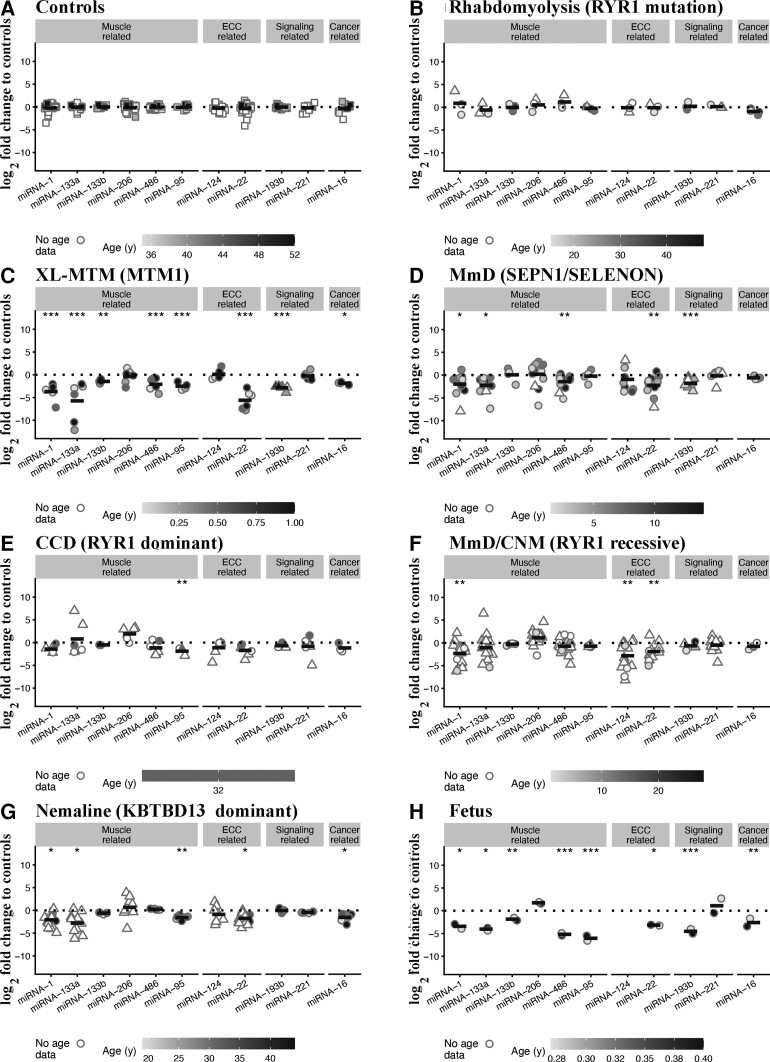
**Muscles from patients with congenital myopathies show significant changes in the expression levels of miRNAs targeting transcripts encoding proteins involved in ECC, muscle, epigenetic and signalling-related transcripts.** Expression levels of the indicated transcripts were determined by qPCR and normalized to the expression of U6. Muscle biopsies were from: (**A**) healthy controls; (**B**) patients with exertional rhabdomyolysis/heat stroke/exercise intolerance carrying *RYR1* mutations; (**C**) patients with *MTM1-*related XL-MTM; (**D**) patients with AR *SELENON*-related MmD; (**E**) patients with AD *RYR1*-related CCD; (**F**) patients with AR *RYR1*-related MmD/CNM; (**G**) patients with AD *KBTBD13-*related nemaline myopathy; (**H**) foetuses. The greyscale given to the symbols reflects the age range of the patients and the scale bar at the bottom of each panel correlates greyscale to age. Empty symbols represent patients or probands whose age was not known. Square symbols represent results from controls; circles represent results from disease patients and foetuses obtained in the present study; triangles represent data obtained from previous investigations.^19,21,23^ The relative transcript expression in patient muscles was compared with that in muscles from healthy controls that was set to 1. Statistical analysis was performed using ‘R’ version 4.21.0 running on platform x86_64-apple-darwin13.4.0 (64 bits). Comparisons of each disease group (or foetus) to controls were calculated using the limma package^24^ of ‘R’. Obtained *P*-values were adjusted for multiple testing using Benjamini–Hochberg method to control the false discovery rate. Means were considered statistically significant when the adjusted *P*-values were <0.05. The horizontal black bar represents the mean content levels in patient muscles. *P < 0.05; **P < 0.01; ***P < 0.001.

To date more than 2000 miRNAs that have been identified in humans and their expression levels are dysregulated in disorders including cancer, ALS, MmD and muscular dystrophies.^23,44–47^ We next investigated miRNA expression levels in muscles from foetuses, disease controls and CM patients and compared them to the expression in muscles from healthy controls (the latter were set to 1, Fig. 5A). We chose to quantify the expression of myo-miRNAs, i.e. those that are specifically expressed in muscles including miRNA-1, -95, -133, -206 and -486,^48^ miRNAs that are predicted to bind to the 3′-UTR of the *RYR1* (miRNA-22 and -124) and to bind to *HDACs* (epigenetic related), and miRNAs binding to transcripts whose products are involved in signalling (miRNA-19 and 221) and tumour suppression/proliferation (cancer related, miRNA-16). Because of the lack of sufficient biological material for the groups of MmD/CNM, CCD, XL-MTM, nemaline and *SEPN1* patients, we added data from our previously published results,^19,21,23^ in order to increase sample sizes (data from previously published results are shown as triangles in Fig. 5). Similar to gene expression results, muscles from disease controls, i.e. patients with *RYR1*-related rhabdomyolysis/exercise intolerance showed no changes in expression of any of the investigated miRNAs (Fig. 5B). On the other hand, miRNA expression levels were disrupted in muscles from all CM patients. Indeed, muscles from XL-MTM patients exhibited the greatest changes in miRNA expression levels, showing significantly lower expression levels of miRNA-1, -95, -133a, -133b, -486, -22, -193b and -16 (the mean log_2_-fold changes were −3.44 adjusted *P* = 9.03E − 05, −2.42 adjusted *P* = 6.53E − 06, −5.66 adjusted *P* = 6.53E − 06, −1.42 adjusted *P* = 0.002, −2.42 adjusted *P* = 9.92E − 04, −5.27 adjusted *P* = 1.24E − 10, −2.81 adjusted *P* = 1.34E − 12 and −1.50 adjusted *P* = 0.01, respectively; Fig. 5C). Muscles from AR *SELENON*-related MmD patients showed significantly decreased expression of miRNA-1, -133a, -486, -22 and -193b (the mean log_2_-fold changes were −1.73 adjusted *P* = 0.01, −2.17 adjusted *P* = 0.02, −1.39 adjusted *P* = 0.005, −1.93 adjusted *P* = 0.003 and −1.74 adjusted *P* = 9.79E − 09, respectively; Fig. 5D). In muscles from AD-*RYR1*-related CCD patients, expression levels of miRNA-95 were significantly decreased (the mean log_2_-fold change was −1.80 adjusted *P* = 0.006; Fig. 5E). Muscles from AR *RYR1*-related MmD/CNM patients showed significantly decreased expression levels of miRNA-1, -22 and -124 (the mean log_2_-fold changes were −2.06 adjusted *P* = 0.003, −1.58 adjusted *P* = 0.04 and −2.57 adjusted *P* = 0.003, respectively; Fig. 5F). Muscles from AD nemaline myopathy patients showed significantly decreased expression levels of miRNA-1, -95, 133a, -22 and -16 (the mean log_2_-fold changes were −1.82 adjusted *P* = 0.01, −1.49 adjusted *P* = 0.003, −2.73 adjusted *P* = 0.01, −1.41 adjusted *P* = 0.01 and −1.17 adjusted *P* = 0.01, respectively; Fig. 5G). Foetal muscles showed a significant decrease in the expression of all miRNA investigated except miRNA-206 and miRNA-221 that were unchanged (Fig. 5H). MiRNA-124 was not detected in foetal muscles (see Supplementary Table 11 for the complete data set and statistical analysis).

In conclusion, (i) the expression levels of most myo-miRNAs and miRNA-22 are downregulated in muscles of all CM patients; (ii) miRNA-95 expression levels are significantly decreased in CM patients carrying dominant *RYR1* mutations; and (iii) foetal muscles show altered expression levels of most of the miRNAs that were investigated.

## Discussion

In this study, we performed a targeted transcriptome analysis on muscle biopsies from 50 patients with genetically diverse muscle disorders. We focused on the expression levels of transcript encoding proteins involved in important aspects of skeletal muscle function including ECC, Ca^2+^ homeostasis, contractile and sarcomeric proteins and regulation of transcription, in order to identify (i) variations in gene expression that may contribute to the development of muscle weakness observed in all these conditions (i.e. generic targets) and (ii) disease-specific transcriptional changes (i.e. disease-specific targets). Our results show that those disorders which impact muscle function more strongly are accompanied by an overall change in expression of a multitude of transcripts. On the other hand, in muscles from mildly affected individuals such as the disease control group, the expression levels of transcripts and miRNAs were almost indistinguishable from those of the healthy control group. This is an important finding since disease controls carry mutations in the *RYR1* gene resulting in hypersensitivity to RyR1 agonists, but are otherwise healthy and do not exhibit a weak muscle phenotype.

Our results show that the expression levels of transcripts encoding many proteins important for muscle function, development and miRNAs (generic targets) are significantly altered in muscles from all CM patients. In particular, muscles from all CM patients investigated showed some commonly mis-regulated transcripts, including reduced levels of *RYR1*, *ATP2B2*, miRNA-1, miRNA-22, increased levels of *DNMT* and Class II *HDACs*. *RYR1* encodes the RyR1 sarcoplasmic reticulum Ca^2+^ channel one of the key proteins involved in skeletal muscle ECC. Decreased RyR1 protein content has been reported in muscles from patients with AR *RYR1* and *SELENON*-related MmD and *MTM1-*related XL-MTM^19–23^ but not in patients with nemaline myopathy. In line with previous findings, *RYR1* expression was either unchanged or reduced in muscles of CCD patients carrying dominant *RYR1* mutations.^49,50^ This variable expression may be linked to the type of mutation present in the proband, which may influence the stability of the transcript. It should also be mentioned that most CCD-linked *RYR1* mutations strongly influence the biophysical properties of the RyR1 calcium channel resulting in less calcium released during ECC.^51,52^ The reduced amount of RyR1 protein and/or the presence of mutant channels is expected to induce lower amounts of calcium released during ECC and consequently to weaker muscles.

*ATP2B2* encodes for the plasma membrane Ca2+ATPase (PMCA) a pump involved in extruding Ca^2+^ ions from the myoplasm to the extracellular environment.^31^ The observed decreased expression of *ATP2B2* may be a compensatory mechanism of the muscle to the lower levels of myoplasmic calcium achieved during ECC consequent to the reduced RyR1 calcium channels. In line, Cully *et al*.^53^ reported that muscles from *mdx* mice that show increased levels of cytosolic calcium, exhibit increased levels of PMCA levels. Nevertheless, the overall impact of reduced PMCA expression is difficult to decipher, especially since skeletal muscles express other calcium pumps (SERCAs) and transporters (Na/Ca exchanger) involved in regulating the myoplasmic calcium concentration. A reduction in PMCA content would decrease the extrusion rate of calcium from the myoplasm into the extracellular environment and this may affect the open probability of the RyR1, since its Po is decreased at high-calcium concentrations.^54^

Interestingly, transcripts encoding the three InsP3R calcium channel isoforms namely *ITPR1*, *ITPR2* and *ITPR3* were elevated in muscle biopsies from many CM patients. Increased *ITPR* expression was reported in muscles from patients with AD and AR *RYR1* CM.^49,55^ Even though InsP3Rs are calcium channels, they cannot functionally replace the RyR1 because of their different biophysical properties, mode of activation (they are ligand-gated channels and are physiologically activated by the second messenger inositol 1,4,5-trisphosphate), the fact that they are not coupled to the voltage sensing dihydropyridine receptor and most likely not located in the junctional face membrane. Remarkably, Suman *et al*.^55^ showed that in myotubes from patients carrying *RYR1* mutations, there is an inverse correlation between RyR1 content and (pan)InsP3R content. However, no insight into the mechanism leading to the switch in expression of these intracellular Ca^2+^ channels was provided and it is reasonable to hypothesize that it may be partially caused by the presence of a greater number of immature muscle cells in patient-derived myotube cultures.

On the other hand, the underlying cause leading to the expression of InsP3R may be linked to the lack of Ca^2+^ signals originating from fewer or mutated RyR1 Ca^2+^ channels which in turn may impact the muscle proteostatic machinery, leading to the aberrant expression of many proteins. Indeed, (i) in the absence of RyR1, mouse muscles aberrantly express >300 genes, including atypical expression of transcription factors, contractile proteins, muscle-specific structural proteins, and muscle regulatory factors^56^; (ii) *RYR1* silencing induces a decrease in myotube area and fusion index^57^; (iii) pharmacologically blocking RyR1 activity inhibits *in vitro* differentiation of foetal myoblasts^58^; (iv) muscles from XL-MTM patients express the lowest levels of *RYR1* and show the greatest changes in overall transcript expression; and (v) expression of the MyHC-EO isoform during development is dependent on the function of the vestibular system and when reduced amounts of mutant RyR1 channels are present, extraocular muscles fail to express MyHC-EO.^59,60^ Hence, it is reasonable to assume that calcium release mediated by RyR1 channels also regulates gene expression. Aside its indirect influence on the expression of myogenic factors such as Pax7, MyoD, MyoG and Mrf4, transcription factors and signalling molecules,^56^ we hypothesize that RyR1-mediated calcium signals impact the accessibility of chromatin to transcription by influencing epigenetic enzymes such as HDACs and DNMT. HDACs can be subdivided into three main groups of which Subgroups IIa (HDAC-4, -5, -7 and -9) and IIb (HDAC-6 and -10) exhibit tissue specificity and are highly expressed in brain, heart and skeletal muscle.^39,61^ When present in the nucleus, HDAC-4 and -5 directly bind and sequester MEF2 thereby repressing its transcriptional activity.^41,42^ Phosphorylation of HDAC-4 by calcium calmodulin–dependent protein kinase (CamK) promotes its export into the cytosol thereby de-repressing the transcription of MEF2-dependent genes.^41^ The physiological validity of this pathway which provides a direct link between calcium homeostasis and HDAC function was demonstrated in mouse cardiomyocytes. In this cellular model, KCl-induced depolarization (which causes a cytoplasmic calcium transient) leads to the export of HDAC-4 and -5 from the nucleus into the cytoplasm and to increased acetylation of histone H3 and H4.^62^ The transcriptional repressing activity of HDACs is strengthened by DNA methylating enzyme (DNMT1, DNMT2/TRDMT1 and DNMT3) such that de-acetylated histones preferentially accumulate on hypermethylated DNA.^63^

Thus, gene transcription is finely controlled within tissues and cells and its regulation occurs at different hierarchical levels, including chromatin accessibility and the presence of specific transcription factors. An additional level of post-transcriptional control is provided by miRNAs which preferentially bind to 3′-UTR of a transcript leading to its degradation. One miRNA can bind to several targets and the 3′-UTR of one transcript can bind several miRNAs thereby creating an intracellular epigenetic circuit.^44^ DNA methylation and histone modifications regulate the expression of various miRNAs^64^ (including miRNA-124, -137 and -148) and a multitude of miRNAs target HDACs and DNMTs (including miRNA-206, -1 and -95). The potential role of such an epigenetic circuit in CM is exemplified by the fact that 26 miRNAs are predicted to target *RYR1* including miRNA-124 and -22 and miRNA-22 also binds to the 3′-UTR of *HDAC4* (http://www.mirdb.org/cgi-bin/search.cgi). Thus, decreased content of miRNA-22 may lead to increased levels of *HDAC4*. Furthermore, miRNA-206 targets >100 transcripts including *DNMT3a*, *MEF2D* and *HDAC4*^65^ (http://www.mirdb.org/mirdb/index.html). Our results clearly show that miRNA expression is altered in human muscles carrying genetically diverse mutations, but the functional significance of these changes is difficult if not impossible to determine and will require more in depth investigations.

In the present study, we also analysed transcript expression in foetal muscles and our results reveal similarities in RNA expression between foetal muscles and muscles from patients affected by CM, especially younger patients affected by XL-MTM. This observation deserves to be investigated in the future and may shed important clues as to developmental abnormalities of muscles caused by the primary genetic defects. Of interest, compared with mature healthy muscles, foetal muscles exhibit low expression levels of *RYR1* and high levels of *ITPR1*, *ITPR2*, *ITPR3*, *STIM1*, *DNMT3A* and of all HDACs (except *HDAC4*) as well of transcription factors belonging to the *NFAT* and *MEF2* families.

## Conclusion

This study provides important insight into the changes occurring in muscles of patients with genetically diverse CM. From a global point of view, muscles from patients with *RYR1* mutations associated with rhabdomyolysis were similar to controls, whereas muscles from XL-MTM patients showed the greatest changes in gene expression, affecting almost all of the transcripts and miRNAs investigated. Thus, the lack of the lipid phosphatase myotubularin 1 dramatically influences the overall composition of mature skeletal muscles. We are aware of the limitations of qPCR-based approaches, and particularly that they do not provide a global overview of all transcriptional changes occurring in a tissue. In addition, the utility of our data in a clinical setting needs to be further developed before it could be used to extrapolate clinical biomarkers. Nevertheless, our study provides a snapshot of the changes occurring at a specific timepoint during disease and presents important information as to the correlation between particular types of mutations and gene expression. The results of this study support the idea for developing cross therapies for patients with genetically diverse CM. Furthermore, our results may provide useful information in the future, for clinicians monitoring transcript expression in patients enrolled in clinical trials using drugs to improve muscle function.

## Supplementary Material

fcac224_Supplementary_Data

## Abbreviations

AD =: autosomal dominant
AR =: autosomal recessive
CCD =: central core disease
CM =: congenital myopathies
CNM =: centronuclear myopathy
DNMT =: DNA methyl transferase
ECC =: excitation–contraction coupling
HDAC =: histone deacetylase
MmD =: multiminicore disease
MH (S) =: malignant hyperthermia (susceptibility)
miRNA =: microRNA
MyHC =: myosin heavy chain
PMCA =: plasma membrane Ca2^+^ATPase
RyR1 =: ryanodine receptor 1
SERCA =: sarcoplasmic reticulum Ca2^+^ATPase
