# Supplementary material for: Targeted transcript analysis in muscles from patients with genetically diverse congenital myopathies
Source: Brain Commun. 2022 Sep 2;4(5):fcac224. doi: 10.1093/braincomms/fcac224 (PMC9525005; doi:10.1093/braincomms/fcac224)
Supplement: fcac224_Supplementary_Data [file fcac224_supplementary_data.docx]

**Supplementary Table 1:** List of gene transcripts analyzed and summary of the function of the protein.

| **ECC and calcium homeostasis** | | **Contractile and sarcomeric** | | **Epigenetic enzymes** | | **Transcription factors and splicing** | |
| --- | --- | --- | --- | --- | --- | --- | --- |
| Gene name | Protein name and function | Gene name | Protein name and function | Gene name | Protein name and function | Gene name | Protein name and function |
| *RYR1* | RyR1.^1^ calcium release channel of the sarcoplasmic reticulum | *MYH1* | MyHC type 2X.^11^ Mainly found in fast glycolytic muscles. | *DNMT1* | DNA methyltransferase 1 (DNMT1). Mainly involved in maintenance of DNA methylation. ^13^ | *NFATC2* | Nuclear factor of activated T-cells 2.^15^ Transfers in a Ca^2+^ dependent fashion from the cytosol to the nucleus where it acts as a transcription factor. Important for muscle differentiation and maturation. |
| *CACNA1S* | Ca_v_1.1.^2^ alpha 1 subunit of the voltage sensor dihydropyridine receptor on T-tubules. | *MYH2* | MyHC type 2A.^11^ Mainly found in intermediate oxidative/ glycolytic muscles. | *TRDMT1* | t-RNA methyltransferase or DNA methyltransferase 2. Mainly involved in methylation of Asp tRNA.^13^ | *NFATC3* | Nuclear factor of activated T-cells 3.^15^ Transfers in a Ca^2+^ dependent fashion from the cytosol to the nucleus where it acts as a transcription factor. Important for muscle differentiation and maturation |
| *STAC3* | SH3 and cysteine rich domain 3.^3, 4^ complexes with Cav1.1 and stabilizes it on the T-tubules | *MYH7* | MyHC type I.^11^ Mainly found in slow twitch oxidative muscles. | *DNMT3A* | DNA methyltransferase 3A (DNMT3a). Mainly involved in de novo DNA methylation. ^13^ | *MEF2A* | Mef2a.^16,17^ Transcription factor involved in the control of muscle gene expression. Complexes with class II HDACs resulting in decrease transcription of mef2 dependent genes. |
| *ATP2A1* | SERCA1.^5^ SR calcium pump of fast twitch muscles. | *ACTN2* | α-actinin 2. Anchors actin thin filaments to titin and Z-discs. | *HDAC1* | Class I histone de-acetylase, located in the nucleus and ubiquitously expressed; repress transcription.^14^ | *MEF2C* | Mef2c.^16,17^ Transcription factor involved in the control of muscle gene expression. Complexes with class II HDACs resulting in decrease transcription of mef2 dependent genes. |
| *ATP2A2* | SERCA2.^5^ SR calcium pump of slow twitch muscles. | *TTN* | Titin. Essential component of the sarcomere. Connects Z-disc to M-line. | *HDAC3* | Class I histone de-acetylase, mostly located in the nucleus and expressed in many cell types; represses transcription ^14^ | *MEF2D* | Mef2d.^16,17^ Transcription factor involved in the control of muscle gene expression. Complexes with class II HDACs resulting in decrease transcription of mef2 dependent genes. |
| *CASQ1* | calsequestrin 1^5^: Calcium buffering protein localized in the lumen of the SR | *TNNT1* | Slow muscle troponin T (sTnT). Binds to tropomyosin and positions it on actin; modulates muscle contraction. | *HDAC4* | Class IIa histone de-acetylase, shuttles between the cytoplasm and the nucleus; are mainly expressed in heart, brain and skeletal muscle; bind MEF2; represstranscription.^14^ | *MBNL1* | Muscleblind-like protein 1 (Mbnl1).^18^ Mediates pre-mRNA alternative splicing regulation. |
| *ATP2B2* | Plasma Membrane CaATPase.^6^ Involved in regulating the resting Ca^2+^ concentration | *TNNT3* | Fast muscle troponin T (fTnT). Binds to tropomyosin and positions it on actin; modulates muscle contraction. | *HDAC5* | Class IIa histone de-acetylase, shuttle between the cytoplasm and the nucleus; are mainly expressed in heart, brain and skeletal muscle; bind MEF2; repress transcription.^14^ |  |  |
| *STIM1* | Stromal interactive molecule 1.^7^ Interacts with Orai1 to promote Ca^2+^ influx under conditions of Ca^2+^store depletion | *MYOM2* | Myomesin 2 (protein M).^12^ Stabilize the 3D protein arrangement of the M-band. | *HDAC9* | Class IIa histone de-acetylase, shuttle between the cytoplasm and the nucleus; are enriched in heart, brain and skeletal muscle; bind MEF2; repress transcription.^14^ |  |  |
| *ORAI1* | Orai1.^7^ Interacts with Stim1 to promote Ca^2+^ influx under conditions of Ca^2+^store depletion |  |  |  |  |  |  |
| *HOMER2* | homer2.^8,9^ An adaptor protein that physically interact with and regulates the gating properties of the RyR1 and InsP3R |  |  |  |  |  |  |
| *ITPR1* | InsP3R1.^10^ calcium channels of the endoplasmic reticulum |  |  |  |  |  |  |
| *ITPR2* | InsP3R2.^10^ calcium channels of the endoplasmic reticulum |  |  |  |  |  |  |
| *ITPR3* | InsP3R3.^10^ calcium channels of the endoplasmic reticulum |  |  |  |  |  |  |
|  |  |  |  |  |  |  |  |

**Supplementary Table 2:** Comparison of CT values for DES and GAPDH in muscle biopsies from controls, and patients carrying AD-*RYR1* and *SEPN1* mutations

| **Genotype** | ***DES*** | | ***GAPDH*** | |
| --- | --- | --- | --- | --- |
|  | **CT Value** | **Mean** | **CT Value** | **Mean** |
| control | 16.9181 | **16.5234** | 16.8539 | **16.5303** |
| control | 16.4681 |  | 15.8645 |  |
| control | 16.4287 |  | 16.4392 |  |
| control | 16.2785 |  | 16.9635 |  |
| CCD patient | 17.8684 | **17.0353** | 18.3787 | **18.1722** |
| CCD patient | 16.4256 |  | 18.3397 |  |
| CCD patient | 16.8118 |  | 17.7982 |  |
|  |  | | | |
| **Genotype** | ***DES*** | | ***GAPDH*** | |
|  | **CT Value** | **Mean** | **CT value** | **Mean** |
| control | 18.5556 | **17.9590** | 19.4502 | **19.0019** |
| control | 16.8968 |  | 18.3836 |  |
| control | 17.2418 |  | 18.7062 |  |
| control | 18.5594 |  | 19.1432 |  |
| control | 18.5412 |  | 19.3263 |  |
| SEPN1 patient | 21.6313 | **19.9793** | 21.4619 | **20.4348** |
| SEPN1 patient | 20.3502 |  | 20.3257 |  |
| SEPN1 patient | 19.0070 |  | 19.9521 |  |
| SEPN1 patient | 19.2184 |  | 19.9976 |  |
| SEPN1 patient | 19.6893 |  | 20.4369 |  |

**Supplementary Table 3:** Sequences of primers used for qPCR

| **Gene** | **Forward** | **Reverse** |
| --- | --- | --- |
| *DES housekeeping gene* | 5’-GGATGGGGAGGTCGTCAGT-3’ | 5’-CTGGGATGGAAGAAGGCTGG-3’ |
| *GAPDH* | 5’-CTGCACCACCAACTGCTTAGC-3’ | 5’-GGCATGGACTGTGGTCATGAG-3’ |
| *RYR1* | 5’-GGACCTCTACGCCCTGTATC-3’ | 5’-ATCTCCCGCCTTAGCCATTTT-3’ |
| *CACNA1S* | 5’-TGTCTTCCTGGGGGTCTTCA-3’ | 5’-GCTCCTTTGCTGCTCATTGG-3’ |
| *STAC3* | 5’- AATGGTGGCGGGGGAAAATC-3’ | 5’- CCCGCTTCGTCTCCTTTCTG-3’ |
| *ATP2A1* | 5’-GGTGCTGGCTGACGACAACT-3’ | 5’-AAGAGCCAGCCACTGATGAG-3’ |
| *ATP2A2* | 5’-CGAACCCTTGCCACTCATCTT-3’ | 5’-GATTTTCAGCACCATCAGCCAC-3’ |
| *ATP2B2* | 5’-CTGCTGTGGGTGTGAACTCT-3’ | 5’- CCGTCCTGTTGTTTGGCTTT-3’ |
| *STIM1* | 5’-CCTTGTCCATGCAGTCCCC-3’ | 5’-CTCGCCCTCTACCAACCTCT-3’ |
| *ORAI1* | 5’-GCTTCGCCATGGTGGCAAT-3’ | 5’-AGAACTTGACCCAGCAGAGC-3’ |
| *CASQ1* | 5’-TGTTACTGATGCGGATAGCGT-3’ | 5’-CAGTGGGGCTGAAAGATGGT-3’ |
| *HOMER2* | 5’-GTGGACGGAGCCAAGGTGA-3’ | 5’-GTGTTGGCTCTGCTGTCGG-3’ |
| *ITPR1* | 5’-ACCACCATCCAGCACTCCTT-3’ | 5’-GCATCCCTGTCTACCTCATCGT-3’ |
| *ITPR2* | 5’-TCATCAGCACCTTGGGGTTAGT-3’ | 5’-TGTGGTTCCCTTGTTTGGCTTG-3’ |
| *ITPR3* | 5’-ACTTTGGGGCTGGTGGATGA-3’ | 5’-TTGAAGAGGCAGTCACGGAAC-3’ |
| *DNMT1* | 5′-AGAACGGTGCTCATGCTTACA-3′ | 5′-CTCTACGGGCTTCACTTCTTG-3’ |
| *TRDMT1* | 5′-TGCCAAGACGATTGAAGGCAT-3′ | 5′-GCAGGGAGGGCTCATTAAAAT-3’ |
| *DNMT3A* | 5’-AGCGGGTTGTGAGAAGGAATG-3’ | 5’-CTTTCTTCTCAGCCGTATCACACT-3’ |
| *HDAC1* | 5’-TCAAGCCGGTCATGTCCAAA-3’ | 5’-CCTCCCAGCATCAGCATAGG-3’ |
| *HDAC3* | 5’-TAATGCCTTCAACGTAGGCGA-3’ | 5’-AGCCAGAGGCCTCAAACTTCT-3’ |
| *HDAC4* | 5’-AGGCTCAGACTTGCGAGAAC-3’ | 5’-ATCTGGTCTCTTTTCGGCGG-3’ |
| *HDAC5* | 5’-GGCAGAAGCTAGACAGCAAG-3’ | 5’-TCCATTCTTGAGCTCTCCTG-3’ |
| *HDAC9* | 5’-CTCTCCACCCCTTAGTGGAAC-3’ | 5’-TTGGGCTCAGAGGCAGTTTT-3’ |
| *NFATC2* | 5’-CGGTGGATAAGGACAAGAGCC-3’ | 5’-GTCTTGATGGCTGGGACTGG-3’ |
| *NFATC3* | 5’-AGGGAAAAATGTCAAGGGGCTC-3’ | 5’-CAGGATGAGGCACAGGCAAA-3’ |
| *MEF2A* | 5’-GCTCTCTCCACCTCAAACCA-3’ | 5’-TGCTCAACATCCCACCTGC-3’ |
| *MEF2C* | 5’-CTG GTG TAA CAC ATC GAC CTC-3’ | 5’-GAT TGC CAT ACC CGT TCC CT-3’ |
| *MEF2D* | 5’-CAGCAGCCAGCACTACAGAG-3’ | 5’-GACGTAGCCATTCCCAACAGG-3’ |
| *MBNL1* | 5’-ACCCGTGCCAATGTTTTCAG-3’ | 5’-GCTTGGAGAAACAGGTCCCA-3’ |
| *ACTN2* | 5’-GACATCGTGAACACCCCTAAAC-3’ | 5’-CCGCAAAAGCGTGGTAGAA-3’ |
| *MYH1* | 5’-GCATCTCTACGCCAGGGTC-3’ | 5’-GAGCAGCCTCCCCAAAAATG-3’ |
| *MYH2* | 5’-AGAAACTTCGCATGGACCTAGA-3’ | 5’-CCAAGTGCCTGTTCATCTTCA-3’ |
| *MYH7* | 5’-TGAGAAGGGCAAAGGCAAGG-3’ | 5’-TCCATCACCCCTGGAGACTT-3’ |
| *MYOM2* | 5’-GCTTGCTTTGCAGGAGTCAG-3’ | 5’-CGTGCTCCCAGACAGAAAGG-3’ |
| *TTN* | 5’-TTTCCACTTCCACTCTGCCC-3’ | 5’-GGTGGTGCTGTCTCAGCTTT-3’ |
| *TNNT1* | 5’-CAGAGAGAGCCGAGCAACA-3’ | 5’-CACGCTTCTGTTCTGCCTTG-3’ |
| *TNNT3* | 5’-GAAACCGAGACCCAAACTCAC-3’ | 5’-AAAGTGGCTGTCGATGAGGG-3’ |

PCR conditions were as follows: 10 minutes at 25°C, 120 minutes at 37°C, 5 minutes at 85°C

qPCR conditions were as follows: 2 minutes at 50°C, 2 minutes at 95°C, 40 x (15 seconds at 95°C, 1 minute at 60°C) using PowerUp^TM^ Sybr^TM^ Green master mix.

**Supplementary Table 4:** miRNA specifications and primer sequences used for miRNA quantification

| **miRNA** | **Human miRBase ID** | **Sequence of primers** |
| --- | --- | --- |
| 1 | hsa-miR-1-3p | 5'-UGGAAUGUAAAGAAGUAUGUAU-3' |
| 16 | hsa-miR-16-5p | 5'-UAGCAGCACGUAAAUAUUGGCG-3' |
| 22 | hsa-miR-22-3p | 5'-AAGCUGCCAGUUGAAGAACUGU-3' |
| 95 | hsa-miR-95-3p | 5'-UUCAACGGGUAUUUAUUGAGCA-3' |
| 124 | hsa-miR-124a-3p | 5'-UAAGGCACGCGGUGAAUGCC-3' |
| 133a | hsa-miR-133a-3p | 5'-UUUGGUCCCCUUCAACCAGCUG-3' |
| 133b | hsa-miR-133b-3p | 5'-UUUGGUCCCCUUCAACCAGCUA-3' |
| 193b | hsa-miR-193b-3p | 5'-AACUGGCCCUCAAAGUCCCGCU-3' |
| 206 | hsa-miR-206-3p | 5'-UGGAAUGUAAGGAAGUGUGUGG-3' |
| 486 | hsa-miR-486-3p | 5'-CGGGGCAGCUCAGUACAGGAU-3' |
| U6  snRNA | housekeeping miRNA | 5'-GTGCTCGCTTCGGCAGCACATATACTAAAATTGG  AACGATACAGAGAAGATTAGCATGGCCCCTGCGCAAGGATGACACGCAAATTCGTGAAGCGTTCCATATTTT-3' |

PCR conditions were as follows: 30 minutes at 16°C, 30 minutes at 42°C, 5 minutes at 85°C

qPCR conditions were as follows: 2 minutes at 50°C, 2 minutes at 95°C, 40 x (15 seconds at 95°C, 1 minute at 60°C) using PowerUp^TM^ Sybr^TM^ Green master mix.

**Supplementary Table 5:** Patient characteristics and general information

| **Sample ID** | **Origin** | **Gender** | **Age at biopsy** | **Mutated gene** | **Disease** | **Gene variants** | **AA Substitution** | **Phenotypic characteristics** |
| --- | --- | --- | --- | --- | --- | --- | --- | --- |
| NL 1074023 | NL | Male | 38 y | *RYR1* AR | Exercise Rhabdo | c.2488C>T c.10219G>A | p.(R830T) p.(A3407T) | Exertional heat stroke episode with rhabdo (CK 440 000) and ICU admission for 6 weeks. IVCT: MHN^§^. No neuromuscular symptoms |
| NL 2336943 | NL | Male | 48 y | *RYR1* AD | Exercise Rhabdo | c.10219G>T | p.(A3407S) | ERM (twice) CK > 500.000; IVCT MHN. |
| D 2427/12 | Germany | Male | 46 y | *RYR1* AD | Exercise Rhabdo | c.7300G>A | p.(G2434R) | ERM, MH reaction^#^, CK 730-2500 U/l, myalgia, fatigue, no paresis.^19, 20^ |
| NL 2654201 | NL | Female | 38 y | *RYR1* AD | Exercise Rhabdo | c.9262G>A | p.(V3088M) | ERM, CK max 60.000 IVCT MHN. |
| D 2725/13 | Germany | Male | 62 y | *RYR1* AD | Exercise Rhabdo | c.6617C>T | p.(T2205M) | ERM, MH reaction^#^, Myoglobinuria, CK 510-6700 U/l, Myalgia, normal findings in neurological examination.^21^ |
| D 3256/16 | Germany | Male | 42 y | *RYR1* AD | Exercise Rhabdo | c.4293G>A | r(spl) | ERM, MH reaction^#^, CK 600 - 2300 U/l, Myalgia, normal findings in neurological examination. |
| D 3379/17 | Germany | Female | 31 y | *RYR1* AD | Exercise Rhabdo | c.7042G>A | p.(E2348K) | ERM, MH reaction^#^, CK 370-755 U/l, Myalgia, normal findings in neurological examination, daughter with similar complaints.^22^ |
| NL S:89015 | NL | Male | 38 y | *RYR1* AD | Exercise Rhabdo | c.1021G>A | p.(G341R) | ERM episode, IVCT: MHS^†^. No additional details available. |
| NL 08-12368 | NL | Female | 15 y | *RYR1* AD | Exercise Rhabdo | c.7300G>A | p.(G2434R) | Normal neuromuscular strength. Suffers from muscle pains and cramps. Has suffered 7 episodes of rhabdo (CK max > 400.000). MHS^† 23^ |
| NL 09-19535 | NL | Male | 20 y | *RYR1* AD | Exercise Rhabdo | c.14545G>A | p.(V4849I) | Normal neuromuscular strength rather bulky muscles and rhabdomyolysis. MHS† |
| NL 11-737 | NL | Male |  | *RYR1* AD | Exercise Rhabdo | c.12862_12900dup | p.(T4288_A4300dup) | ERM, muscle hypertrophy, normal strength. No IVCT data available |
| NL 12-044-04335 | NL | Male | 60 y | *RYR1* AD | Exercise Rhabdo | c.10616G>A | p.(R3539H) | Muscle hypertrophy, myalgia and cramps. Axial muscle weakness |
| NL 14-27216 | NL | Male | 30 y | *RYR1* AD | Exercise Rhabdo | c.14545G>A | p.(V4849I) | Normal neuromuscular strength. No neuromuscular symptoms. Two episodes of ERM CK max > 400 000. MHS^†^ |
| D 2358/11 | Germany | Male | 40 y | *RYR1* AD | CCD | c.1655G>A | p.(R552Q) | CCD, MH reaction^#^, CK 350 U/l, severe congenital myopathy with facial weakness, swallowing problems, respiratory distress, tetraparesis MRC 3-4, severe gait disorder, wheelchair dependent.^24^ |
| BS 01 | CH | Male |  | *RYR1* AD | CCD | Not known | Not known |  |
| NL 08-10960 | NL | Male | 32 y | *RYR1* AD | CCD | c.139470T>C | p.(L4647P) | Difficulties in sports in childhood, mild proximal weakness, ambulant, Achilles contractures. At present, ambulance normal; active in sports, clinically no signs of polyneuropathy. EMG focused on peripheral nerves: no signs of polyneuropathy. Muscle biopsy (age 32): type I predominance, increased variation in fibre diameter, many central cores, Z-band streaming. Patient N° CCD NL 01^25^ |
| SA 1157/09 | SA | Female | 5y | *RYR1* AR | MmD/ CNM | c.11320dupG c. 10348-6C>G c.14524G>A | p.(A3774fs) p.(N3450fs)  p.(V4842M) | Congenital myopathy, reduced in utero movements, sat but remained non-ambulant, proximal weakness, normal intelligence, recurrent respiratory infections, myopathic facies and severe extraocular ophthalmoplegia, scoliosis. Patient N° 4^.26^ |
| I 201203784 | Israel | Male | 1 y | *RYR1* AR | MmD/ CNM | c.12815_12825del  c.9148G>A | p.(A4272fs)  p.(V3050I) | Early onset hypotonia, weakness and feeding difficulties. Non ambulatory, scoliosis. CK 40 IU. Biopsy - centronuclear myopathy. |
| D 2362/11 | Germany | Male | 44 y | *RYR1* AR | MmD/ CNM | c.641C>T c.6617C>T | p.(T214M) p.(T2206M) | ERM, MH reaction^#^, Myopathy, CK 500 – 4000 U/l, myalgia, myopathy with proximal muscle weakness MRC 4, gait problems, severe difficulty to climb stairs. First mutation likely pathogenic, second mutation.^19^ |
| D 2599/13 | Germany | Male | 47 y | *RYR1* AR | MmD/ CNM | c.1840C>T c.538_554del | p.(R614C) p.(H180fs) | ERM, MH reaction^#^, CK 700 - 920 U/l, Myalgia, normal findings in neurological examination, 2 sons carry the second mutation. First mutation^27^ second mutation? Both sons carry this mutation without complaints. |
| D 2878/14 | Germany | Male | 50 y | *RYR1* AR | MmD/ CNM | c.13013_13032del c.14779G>A | p.(A4338fs)  p.(V4927I) | Myopathy, MH reaction^#^, CK 435 U/l, fatigue, myalgia, mild proximal weakness.^28^ |
| Na 01 | Italy | Male | 1 week | *RYR1* AR | MmD/ CNM | c.14928C>G  (homoz.) | p.(F4976L) | Congenital myopathy with severe floppy baby, CK 450 U/I |
| NL 10-19394 | NL | Male | 2 y | *RYR1* AR | MmD/ CNM | c.11905C>A | p.(Q3969K) +second mutation not found | Developmental delay (milestones 4- 5 months delay at age 2 years) and generalized muscle weakness. Facial weakness (open mouth). Axial hypotonia with head lag and slipping through. Gowers sign positive. Hypermobility. Ambulant at latest follow-up at age 6. Biopsy (age 2): increase of central and internal nuclei, type I predominance (67%), on EM several minicores and disruption of Z-lines. Patient N° Minicore NL01.^25^ |
| NL 89-19191 | NL | Male | 28 y | *RYR1* AR | MmD/ CNM | c.4711A>G c.10097G>A c.11798A>G c.14545G>A | p.(I1571V) p.(R3366H) p.(Y3933C) p.(V4849I) | Mild muscle weakness in childhood, gradual increase of muscle weakness. MHS^†^. CK 441 – 1962 U/l. Neurological examination (age 50): mild vertical ophthalmoplegia when looking upwards. Neck flexion, extension 4. Elbow extension and flexion 5. Hands 4. Hip- and knee flexion 4. Can walk on heels, not on toes. Positive Gowers. No joint hypermobility Muscle biopsy (age 28): Fibre type I predominance, hypertrophic fibres, mild increase of endomysial connecetive tissue. Several cores in type I-fibres, also at the peripheral zones of the cells. Increase of internal nuclei. Patient N° Minicore NL03.^25^ |
| NL 3508 | NL | Male | 19 months | *SELENON* AR | MmD | c.713dup c.1332_1334del | p.(N238fs) p.(N444del) | Motor delay. Currently walks up to 100meters. Scoliosis with need for surgery. Respiratory muscle weakness, nocturnal noninvasive mechanical ventilation since age of 10y.  Cognition normal. |
| UK 3866 | UK | Male | 6 y | *SELENON* AR | MmD | 1282-2A>C Homozygous Splice acceptor site | p.(0) | Motor delay with sitting at 9 months and walking holding at 1.5 years of age. Recurrent chest infections in the first few years and heart failure due to sleep hypoventilating at age 16 (he was initiated on cuirass jacket ventilation for 2 years then changed to BIPAP). Scoliosis first noticed at age 10 and surgery performed at age 15.  Currently walking but limited distance. Patient N° 2.^29^ |
| UK 5314 | UK | Female | 13 y | *SELENON* AR | MmD | c.1384T>G (homoz.) | p.(U462G) | Diagnosed at 14 months with axial hypotonia and forward flexed posture on sitting. Through childhood developed spinal rigidity with scoliosis. At the age of 12 referred due to symptoms of severe nocturnal hypoventilation, managed with nocturnal BIPAP. Underwent spinal fusion aged 17 years for progressive scoliosis. Patient N° 4.^29^ |
| UK 6508 | UK | Male | 12 y | *SELENON* AR | MmD | c.1315C>T (homoz.) | p.(R439*) | Delayed motor milestones, right from the first 2 years of life with inability to jump, hop or run, but maintained ability to walk on level ground and going upstairs. Axial proximal facial weakness, contractures and some distal laxity present at age 15.  Nocturnal ventilation since the age of 13 years. Obese phenotype. Patient N° 6.^29^ |
| UK 7220 | UK | Male | 6 y | *SELENON* AR | MmD | c.1A>G c.883G>A | p.M1? p.(E295K) | Progressive muscle weakness. Lumbar lordosis and Rigidity of spine. Recurrent respiratory infections since the age of 9 months. Nocturnal BiPAP from age 5. Patient N° 3.^29^ |
| UK 7264 | UK | Male | 2 y | *SELENON* AR | MmD | c.1282-2A>C (homoz.) | p.(0) | First concern aged 9 months with axial and proximal muscle weakness, and dropped head. Early scoliosis initially noticed aged 2, and frequent falls since acquisition of de-ambulation at 19 months. Patient N° 5.^29^ |
| Den 01 | Denmark | Female | 14 y | *SELENON* AR | MmD | c.446dupC c.943G>A | p.(D150*) p.(G315S) | 24-hr ventilator, rigid spine, spine operation age 30 years. Wheelchair bound since age 25. Patient N° 11.^29^ |
| Den 03 | Denmark | Female | 10y | *SELENON* AR | MmD | c.943G>A | p.(G315S) | Nocturnal ventilator, rigid spine, wheelchair user, but can walk small distances. Patient N° 13.^29^ |
| Den 02 | Denmark | Female | 7 y | *SELENON* AR | MmD | c.893T>C c.1396C>T | p.(L298P) p.(R466W) | Nocturnal ventilator, rigid spine, scoliosis operation age 19, wheelchair user, but can walk small distances. Patient N° 12.^29^ |
| BOS | USA |  | 3 y | *SELENON* AR | MmD | c.1A>G c.943G>A | p.M1? p.(G315S) | 6 year female w/ moderate hypotonia at birth, delayed motor milestones, dysarthria. Currently ambulant for short distances, uses manual wheelchair for longer. On nocturnal ventilation by BiPAP. Patient N° 15.^29^ |
| NL 22413 | NL | Male | 13 y | *SELENON* AR | MmD | c.943G>A c.1332_1334del | p.(G315S) p.(N444del) | Delayed motor development, hypotonia and weakness, respiratory weakness for which no non-invasive nocturnal ventilation yet at 19 years of age, limited walking distance (100 m), scoliosis. Patient N° 9^.29^ |
| NL 00682 | NL | Female | 4 y | *SELENON* AR | MmD | c.713dup c.1332_1334del | p.(N238fs) p.(N444del) | Delayed motor development, hypotonia and weakness, respiratory weakness for which non-invasive nocturnal ventilation since 10 years of age, permanent wheelchair use since at age 27. Scoliosis operation at age 10. Patient N° 7.^29^ |
| NL 05244 | NL | Female | 2 y | *SELENON* AR | MmD | c.943G>A (homoz.) | p.(G315S) | Delayed motor development, hypotonia and weakness, respiratory weakness for which non-invasive nocturnal ventilation since 16 years of age, intermittent wheelchair use since age 19. Scoliosis operation. Patient N° 8.^29^ |
| Den 05 | Denmark | Female | 2 y | *SELENON* AR | MmD | c.713dup c.943G>A | p.(N238fs) p.(G315S) | Proximal weakness and walking distance of 200 m. Rigid spine and scoliosis. 65% FVC |
| NL 09-10405 | NL | Female | 19 y | *KBTBD13* AD | Nemaline myopathy | c.1222C>T | p.(R408C) | Age at onset symptoms: childhood; exercise intolerance; muscle slowness not reported; no cardiac abnormalities, no respiratory difficulties, Proximal muscle weakness; no quadriceps muscle weakness; neck flexion: MRC 2.^30^ |
| NL 09-13176 | NL | Female | 42 y | *KBTBD13* AD | Nemaline myopathy | c.1222C>T | p.(R408C) | Age at onset symptoms: childhood; exercise intolerance; muscle slowness +; cardiac involvement: (ventricular tachycardia; ICD); no respiratory difficulties; limb girdle pattern of muscle weakness, mild quadriceps muscle weakness; neck flexion: MRC 2.^30^ |
| NL 11-9850 | NL | Male | 31 y | *KBTBD13* AD | Nemaline myopathy | c.1222C>T | p.(R408C) | Age at onset symptoms: childhood; no exercise intolerance; muscle slowness +; cardiac abnormalities not reported; no respiratory difficulties; mild muscle weakness.^30^ |
| NL 11-9851 | NL | Female | 28 y | *KBTBD13* AD | Nemaline myopathy | c.1222C>T | p.(R408C) | Age at onset: childhood; exercise intolerance; muscle slowness +; no cardiac abnormalities; no respiratory difficulties; mild muscle weakness, no quadriceps muscle weakness; neck flexor muscle: MRC 3.^30^ |
| NL 12-26869 | NL | Female | 44 y | *KBTBD13* AD | Nemaline myopathy | c.1222C>T | p.(R408C) | Age at onset symptoms: 30s; no exercise intolerance; muscle slowness +; cardiac abnormality: + (cardiac arrythmia; ICD); respiratory difficulties not reported; proximal muscle weakness; neckflexion MRC 3; no quadriceps muscle weakness.^30^ |
| NL 16-4515 | NL | Female | 33 y | *KBTBD13* AD | Nemaline myopathy | c.1222C>T | p.(R408C) | Age at onset symptoms: infancy; no exercise intolerance; muscle slowness +; no cardiac abnormalities; no respiratory difficulties; scapula alata; proximal muscle weakness; no quadriceps muscle weakness; neckflexion MRC 3.^30^ |
| UK 6212 | UK | Male | 3 days | *MTM1* X-linked | XL-MTM | c.1171T>G | p.(Y198*) | Born at 35 weeks gestation, floppy and ventilated at birth; mild facial weakness, swallowing difficulties; proximal distal muscle weakness |
| UK 6499 | UK | Male | 9 days | *MTM1* X-linked | XL-MTM | c.1509_1510del | p.(N503fs) | Born at 41+6 weeks gestation. Antenatally reduced foetal movements; hypotonia since birth; proximal and distal weakness and feeding difficulties requiring NG feeds. Mild low facial weakness and contractures of scapulo humeral joints, elbow, fingers, knees and hips. |
| UK 7457 | UK | Male | 3 months | *MTM1* X-linked | XL-MTM | del 5' part of gene, incl. exon 1 | p.0 | Oxygen dependence at birth; long term ventilation with tracheostomy; facial weakness and ophthalmoplegia, undescended testes, bilateral dislocated hips. |
| UK 13-0078 | UK | Male | 1 y | *MTM1* X-linked | XL-MTM | c.594C>G | p.(Y198*) | Born at 37+2 weeks gestation. Neonatal hypotonia and ventilator dependent, dysmorphic features and nasogastric tube feeding. Bulbar dysfunction; hypotonia more pronounced on trunk, upper limbs worse than lower limbs. |
| UK 14-1816 | UK | Male | 6 months | *MTM1* X-linked | XL-MTM | c.721C>T | p.(R241C) | Severe weakness, proximal orobulbar dysfunction, ophthalmoplegia, laryngomalacia; feeding difficulties, needs endotracheal ventilator. Overall course of gradual improvement hypotonic but has good antigravity power. |
| UK 15-0805 | UK | Male | 1 y | *MTM1* X-linked | XL-MTM | c.1084_1087del | p.(D362fs) | Antenatal decreased foetal movements and polyhydramniosis. Floppiness since birth, ventilator dependent from day 1. Cannot swallow saliva, undescended testes. Facial weakness, legs more than arms, proximal more than distal weakness. Mild leg contractures. |
| 1929959 |  | Male | 33 y | No mutation | Control | - | - | - |
| 1439587 |  | Male | 24 y | No mutation | Control | - | - | - |
| 3551-18 |  | Female | 65 y | No mutation | Control | - | - | - |
| 3612-18 |  | Male | 35 y | No mutation | Control | - | - | - |
| 3644-18 |  | Male | 38 y | No mutation | Control | - | - | - |
| 3665-18 |  | Male | 52 y | No mutation | Control | - | - | - |
| 3944-19 |  | Female | 45 y | No mutation | Control | - | - | - |
| 3956-19 |  | Female | 52 y | No mutation | Control | - | - | - |
| 5430681 |  | n.a. | n.a. | No mutation | Control | - | - | - |
| 4420232 |  | n.a. | n.a. | No mutation | Control | - | - | - |
| 4463365 |  | n.a. | n.a. | No mutation | Control | - | - | - |
| 2139250 |  | n.a. | n.a. | No mutation | Control | - | - | - |

^§^ Diagnosed as MHN after the *in vitro contracture test*. ^#^ Suffered an MH reaction. †Diagnosed as MHS after the *in vitro contracture test*.

**Supplementary Table 6**: Classification of the gene variants identified in the patients.

| **Mutated gene and reference sequence** | **Gene variant** | **AA Substitution** | **Inherit.** | **PubmedID**  **(1st report)*** | **ACMG criteria** | **ACMG classification*** |
| --- | --- | --- | --- | --- | --- | --- |
| *RYR1*  NM_000540.3 | c.538_554del | p.(H180fs) | AR | - | PVS1, PM2 | LP (AR) |
|  | c.641C>T^$^ | p.(T214M) | AD | [33333461](https://pubmed.ncbi.nlm.nih.gov/33333461/) | PP3 | VOUS (AR) |
|  | c.1021G>A^$^ | p.(G341R) | AD | - | PP3 | VOUS (AR) |
|  | c.1655G>A | p.(R552Q) | AD | - | - | VOUS (AR) |
|  | c.1840C>T^$^ | p.(R614C) | AR | [30611313](https://pubmed.ncbi.nlm.nih.gov/30611313/) | PP3 | VOUS (AR) |
|  | c.2488C>T | p.(R830W) | AR | [25960145](https://pubmed.ncbi.nlm.nih.gov/25960145/) | - | VOUS (AR) |
|  | c.4293G>A | r(spl?) | AD |  | PVS1, PM2 | LP (AR) |
|  | c.4711A>G^#^ | p.(I1571V) | AR | [22473935](https://pubmed.ncbi.nlm.nih.gov/22473935/) | - | VOUS (AR) |
|  | c.6617C>T^$^ | p.(T2206M) | AR | [23919265](https://pubmed.ncbi.nlm.nih.gov/23919265/) | PM2, PM3, PP3 | VOUS (AR) |
|  | c.7042G>A | p.(E2348K) | AD | - | PM2, PP3 | VOUS (AD, AR) |
|  | c.7300G>A^$^ | p.(G2434R) | AD | - | PP3 | VOUS (AR) |
|  | c.9148G>A | p.(V3050I) | AR | [32403337](https://pubmed.ncbi.nlm.nih.gov/32403337/) | PM3 | VOUS (AR) |
|  | c.9262G>A | p.(V3088M) | AD | - | PP3 | VOUS (AR) |
|  | c.10097G>A^#^ | p.(R3366H) | AR | [21674524](https://pubmed.ncbi.nlm.nih.gov/21674524/) | - | VOUS (AR) |
|  | c.10219G>A | p.(A3407T) | AR | - | PP3 | VOUS (AR) |
|  | c.10219G>T | p.(A3407S) | AD | - | PM2, PP3 | VOUS (AR) |
|  | c.10348-6C>G | p.(N3450fs) | AR | [18253926](https://pubmed.ncbi.nlm.nih.gov/18253926/) | PVS1, PM2, PM3 | P (AR) |
|  | c.10616G>A | p.(R3539H) | AD | [22473935](https://pubmed.ncbi.nlm.nih.gov/22473935/) | PM3, BS1 | VOUS (AR) |
|  | c.11320dup | p.(A3774fs) | AR | [20839240](https://pubmed.ncbi.nlm.nih.gov/20839240/) | PVS1, PM2, PM3 | P (AR) |
|  | c.11798A>G^#^ | p.(Y3933C) | AR | [22473935](https://pubmed.ncbi.nlm.nih.gov/22473935/) | PP3 | VOUS (AR) |
|  | c.11905C>A | p.(Q3969K) | AD | [25960145](https://pubmed.ncbi.nlm.nih.gov/25960145/) | PS2, PM2, PP3 | LP (AD) |
|  | c.12815_12825del | p.(A4272fs) | AR | [25960145](https://pubmed.ncbi.nlm.nih.gov/25960145/) | PVS1, PM2 | LP (AD) |
|  | c.12862_12900dup | p.(T4288_  A4300dup) | AD | - | - | VOUS (AR) |
|  | c.13013_13032del | p.(A4338fs) | AR | [17538032](https://pubmed.ncbi.nlm.nih.gov/17538032/) | PVS1, PM2 | LP (AR) |
|  | c.13940T>C | p.(L4647P) | AD | [23183335](https://pubmed.ncbi.nlm.nih.gov/23183335/) | PM2 | VOUS (AD) |
|  | c.14524G>A | p.(V4842M) | AR | [18253926](https://pubmed.ncbi.nlm.nih.gov/18253926/) | PP3 | LB (in cis with c.10348-6C>G) |
|  | c.14545G>A^$^ | p.(V4849I) | AD | [12136074](https://pubmed.ncbi.nlm.nih.gov/12136074/) | PM3, PP1, PP3 | VOUS (AR) |
|  | c.14779G>A | p.(V4927I) | AR | - | PP3 | VOUS (AR) |
|  | c.14928C>G | p.(F4976L) | AR | [30611313](https://pubmed.ncbi.nlm.nih.gov/30611313/) | PM3, PP3 | VOUS (AR) |
| *SELENON* NM_020451.2 | c.1A>G | p.M1? | AR | [12192640](https://pubmed.ncbi.nlm.nih.gov/12192640/) | PVS1, PM2 | LP |
|  | c.446dup | p.(D150*) | AR | [30932294](https://pubmed.ncbi.nlm.nih.gov/30932294/) | PVS1, PM2, PM3 | P |
|  | c.713dup | p.(N238fs) | AR | [12192640](https://pubmed.ncbi.nlm.nih.gov/12192640/) | PVS1, PM3 | LP |
|  | c.883G>A | p.(E295K) | AR | [21670436](https://pubmed.ncbi.nlm.nih.gov/21670436/) | PM2, PM3, PP3 | VOUS |
|  | c.893T>C | p.(L298P) | AR | [28357410](https://pubmed.ncbi.nlm.nih.gov/28357410/) | PM2, PP3 | VOUS |
|  | c.943G>A | p.(G315S) | AR | [12192640](https://pubmed.ncbi.nlm.nih.gov/12192640/) | PM2, PM3, PP1, PP3 | LP |
|  | c.1282-2A>C | p.(0) | AR | [21670436](https://pubmed.ncbi.nlm.nih.gov/21670436/) | PVS1, PM2 | LP |
|  | c.1315C>T | p.(R439*) | AR | [15792869](https://pubmed.ncbi.nlm.nih.gov/15792869/) | PVS1, PM3 | LP |
|  | c.1332_1334del | p.(N444del) | AR | [30932294](https://pubmed.ncbi.nlm.nih.gov/30932294/) | PM2, PM3, PP1, PP3 | LP |
|  | c.1384T>G | p.(U462G) | AR | [12192640](https://pubmed.ncbi.nlm.nih.gov/12192640/) | PM2 | VOUS |
|  | c.1396C>T | p.(R466W) | AR | [28357410](https://pubmed.ncbi.nlm.nih.gov/28357410/) | PM2, PP3 | VOUS |
| *KBTBD13* NM_001101362.3 | c.1222C>T | p.(R408C) | AD | [21109227](https://pubmed.ncbi.nlm.nih.gov/21109227/) | PM2, PP1, PP3 | VOUS |
| *MTM1* NM_000252.3 | del 5' part of gene, incl. exon 1 | p.0 | XL |  | PVS1, PM2, PP4 | P |
|  | c.594C>G | p.(Y198*) | XL | [10063835](https://pubmed.ncbi.nlm.nih.gov/10063835/) | PVS1, PM2, PP4 | P |
|  | c.721C>T | p.(R241C) | XL | [9305655](https://pubmed.ncbi.nlm.nih.gov/9305655/) | PM2, PP3, PP4 | VOUS |
|  | c.1084_1087del | p.(D362fs) | XL | [28007904](https://pubmed.ncbi.nlm.nih.gov/28007904/) | PVS1, PM2, PP4 | P |
|  | c.1509_1510del | p.(N503fs) | XL | [22968136](https://pubmed.ncbi.nlm.nih.gov/22968136/) | PM2, PM4, PP4 | VOUS |

*Classification of *RYR1* variant and references in the context of myopathy, not in the context of malignant hyperthermia susceptibility.

# The p.(I1571V), p.(R3366H) and p.(Y3933C) variants have been detected multiple times in recessive myopathy and reside on the same allele (PMID: 25958340), which is considered to be likely pathogenic. ^$^Also associated with malignant hyperthermia susceptibility.

**Supplementary Table 7:** Mean log_2_ fold change in expressionof transcripts encoding proteins involved in ECC and calcium homeostasis in muscles of patients with CM and foetal muscles

|  | **Patient category and mutated gene**  **(mean log2 fold change in expression, P values, adj. P.Values and N samples)** | | | | | |  |
| --- | --- | --- | --- | --- | --- | --- | --- |
| **Gene Target** | Rhabdomyolysis  (AD *RYR1*) | XL-MTM  (*MTM1*) | MmD (*SEPN1/SELENON*) | CCD  AD *RYR1* | MmD  (AR *RYR1)* | Nemaline  (AD *KBTBD13*) | Fetus |
| *RYR1* | 0.1385  P=0.703  adj.P.Val=0.9621  N=7 | -2.756  P=8.02E-09  adj.P.Val=2.89E-08  N=6 | -1.3976  P=1.02E-05  adj.P.Val=6.61E-05  N=13 | -1.582  P=0.0031  adj.P.Val=0.081  N=3 | -1.030  P=0.0060  adj.P.Val=0.0635  N=7 | -1.85  P=9.71E-06  adj. P. Val=8.74E-05  N=6 | -5.561  P=6.13E-16  adj.P.Val=1.10E-14  N=3 |
| *CACNA1S* | 0.1497  P=0.4784  adj.P.Val=0.9621  N=7 | 1.069  P=1.41E-04  adj.P.Val=2.22E-04  N=4 | -1.203  P=1.31E-09  adj.P.Val=2.35E-08  N=13 | 0.114  P=0.7035  adj.P.Val=0.810  N=3 | -0.2313  P=0.2748  adj.P.Val=0.5820  N=7 | 0.0994  P=0.657  adj. P. Val=0.788  N=6 | -0.564  P=0.063  adj.P.Val=0.0992  N=3 |
| *STAC3* | -0.3339  P=0.2258  adj.P.Val=0.852  1N=7 | 1.322  P=2.89E-04  adj.P.Val=4.33E-04  N=4 | -0.891  P=0.0059  adj.P.Val=0.0164  N=5 | 0.589  P=0.131  adj.P.Val=0.493  N=3 | -0.00121  P=0.9965  adj.P.Val=0.996  N=7 | 0.733  P=0.0142  adj. P. Val=0.051  N=6 | -0.336  P=0.385  adj.P.Val=0.4778  N=3 |
| *ATP2A1* | -0.3532  P=0.6102  adj.P.Val=0.9621  N=4 | -3.083  P=4.42E-05  adj.P.Val=7.95E-05  N=4 | -1.958  P=8.59E-04  adj.P.Val=0.00341  N=7 | -4.718  P=1.78E-07  adj.P.Val=3.21E-06  N=3 | -1.4333  P=0.012  adj.P.Val=0.0635  N=7 | -3.953  P=1.48E-08  adj. P. Val=2.67E-07  N=6 | -2.971  P=3.74E-04  adj.P.Val=8.41E-04  N=3 |
| *ATP2A2* | 0.2269  P=0.5746  adj.P.Val=0.9621  N=6 | 0.715  P=0.13  adj.P.Val=0.14  N=4 | -0.62323  P=0.1270  adj.P.Val=0.2285  N=6 | 0.305  P=0.570  adj.P.Val=0.810  N=3 | 0.8851  P=0.0234  adj.P.Val=0.094  N=7 | 0.567  P=0.164  adj. P. Val=0.395  N=6 | -0.534  P= 0.322  adj.P.Val 0.429  N=3 |
| *ATP2B2* | -0.2153  P=0.5968  adj.P.Val=0.9621  N=7 | -3.994  P=4.62E-10  adj.P.Val=2.77E-09  N=4 | -1.4587  P=0.00256  adj.P.Val=0.0077  N=5 | -2.566  P=3.54E-04  adj P. Val=0.0025  N=2 | -1.3455  P=0.0029  adj.P.Val=0.052  N=6 | -0.987  P=0.0254  adj. P. Val=0.0763  N=6 | -1.726  P=0.012  adj.P.Val=0.023  N =2 |
| *STIM1* | 0.211  P=0.3340  adj.P.Val =0.9414  N=7 | 1.306  P=1.54E-05  adj.P.Val=3.07E-05  N=4 | -0.080  P=0.7471  adj.P.Val=0.8480  N=5 | 0.406  P=0.186  adj.P.Val=0.555  N=3 | 0.6228  P=0.00975  adj.P.Val=0.0635  N=6 | 0.0293  P=0.8994  adj. P Val=0.951  N=6 | 1.999  P=1.57E-06  adj.P.Val.=4.34E-06  N=2 |
| *ORAI1* | -0.0129  P=0.9621  adj.P.Val=0.9621  N=4 | 1.443  P=4.16E-06  adj.P.Val=8.82E-06  N=4 | 0.224  P=0.3738  adj.P.Val =0.5606  N=5 | -0.032  P=0.916  adj.P.Val =0.916  N=3 | 0.0205  P=0.9347  adj.P.Val=0.9765  N=5 | 0.151  P=0.5185  adj. P. Val=0.6437  N=6 | -0.239  P=0.516  adj.P.Val= 0.599  N=2 |
| *CASQ1* | 0.3747  P=0.1142  adj.P.Val =0.5934  N=6 | 0.477  P=0.087  adj.P.Val= 0.095  N=4 | 0.2494  P=0.3234  adj.P.Val=0.5062  N=5 | -0.226  P=0.467  adj.P.Val=0.799  N=3 | -0.0758  P=0.7629  adj.P.Val=0.886  N=5 | -0.8459  P=7.04E-04  adj.P.Val =0.003  N=6 | -0.312  P=0.402  adj.P.Val=0.483  N=3 |
| *HOMER2* | -0.1585  P=0.5950  adj.P.Val =0.9621  N=7 | 1.469  P=8.73E-04  adj.P.Val=0.0012  N=3 | 0.03996  P=0.9051  adj.P.Val=0.9583  N=5 | 0.734  P=0.137  adj.P.Val=0.492  N=2 | -0.3995  P=0.3319  adj.P.Val=0.6163  N=3 | 1.325  P=1.35E-04  adj P. Val=8.09E-04  N=6 | 0.724  P=0.142  adj.P.Val=0.213  N=2 |
| *ITPR1* | -0.141  P=0.7116  adj.P.Val=0.962  N=6 | 3.863  P=1.87E-10  adj.P.Val=1.35E-09  N=4 | 1.7370  P=2.97E-04  adj.P.Val =0.00133  N=4 | 0.481  P=0.397  adj.P.Val=0.799  N=2 | 0.3309  P=0.4136  adj.P.Val=0.6473  N=5 | -0.082  P=0.8289  adj.P.Val=0.9042  N=6 | 5.028  P=1.8E-10  adj.P.Val=1.67E-09  N=2 |
| *ITPR2* | -0.0308  P=0.9263  adj.P.Val=0.9621  N=6 | 3.006  P=2.94E-09  adj.P.Val= 1.18E-08  N=4 | 1.0581  P=0.0083  adj.P.Val=0.0213  N=4 | 0.469  P= 0.346  adj.P.Val=0.799  N=2 | 0.5591  P=0.1197  adj.P.Val==0.3590  N=5 | 0.2688  P=0.4222  adj.P.Val=0.5846  N=6 | 3.442  P=4.48E-08  adj.P.Val=1.61E-07  N=2 |
| *ITPR3* | -0.2947  P=0.5662  adj.P.Val=0.9621  N=6 | 2.539  P=9.38E-05  adj.P.Val=1.62E-04  N=4 | 1.0543  P=0.0709  adj.P.Val=0.1595  N=4 | 0.333  P=0.638  adj.P.Val=0.810  N=2 | 0.1870  P=0.7269  adj.P.Val=0.8723  N=5 | -0.4826  P=0.3497  adj.P.Val=0.5247  N=6 | 5.174  P=3.47E-08  adj.P.Val=1.45E-07  N=2 |

Moderated t-statistics was calculated using the limma package.^31^ Obtained P-values were adjusted for multiple testing using Benjamini-Hochberg method to control the false discovery rate

**Supplementary Table 8:** Mean log_2_ fold change in expression of transcripts encoding contractile and sarcomeric proteins, in muscles of patients with CM and foetal muscles

|  | **Patient category and mutated gene**  **(mean log2 fold change in expression, P values, adj. P.Values and N samples )** | | | | | |  |
| --- | --- | --- | --- | --- | --- | --- | --- |
| **Gene Target** | Rhabdomyolysis  (AD *RYR1*) | XL-MTM  (*MTM1*) | MmD (*SEPN1/SELENON*) | CCD  (AD *RYR1*) | MmD  (AR *RYR1*) | Nemaline  (AD *KBTBD13*) | Fetus |
| *ACTN2* | -0.2450  P=0.624  adj.P.Val=0.9621  N=6 | -5.2868  P=8.57E-12  adj.P.Val=1.03E-10  N=4 | 0.9677  P=0.0756  adj.P.Val=0.160  N=5 | 0.4232  P=0.5232  adj.P.Val=0.7990  N=3 | 0.0340  P=0.9493  adj.P.Val=0.9765  N=5 | 0.5688  P=0.2573  adj.P.Val=0.4853  N=6 | -8.9501  P=6.41E-15  adj.P.Val=7.69E-14  N=2 |
| *MYH1* | 0.5700  P=0.4343  adj.P.Val=0.9621  N=6 | 1.520  P=0.0801  adj.P.Val=0.0920  N=4 | -2.7418  P=9.48E-04  adj.P.Val=0.0034  N=5 | -6.43722  P=2.53E-08  adj.P.Val=9.09E-07  N=3 | -0.8985  P=0.2528  adj.P.Val=0.5688  N=5 | -8.1728  P=7.73E-15  adj.P.Val=2.78E-13  N=6 | 0.5020  P=0.6638  adj.P.Val=0.7028  N=2 |
| *MYH2* | -0.0896  P=0.9018  adj.P.Val=0.9621  N=6 | -1.503  P=0.0818  adj.P.Val=0.0920  N=4 | 0.8333  P=0.2869  adj.P.Val=0.4695  N=5 | -4.1353  P=8.10E-05  adj.P.Val=7.29E-04  N=3 | -1.1370  P=0.1485  adj.P.Val=0.4113  N=5 | -3.4450  P=2.16E-05  adj.P.Val=1.55E-04  N=6 | -0.7005  P=0.5390  adj.P.Val=0.6064  N=2 |
| *MYH7* | -0.4232  P=0.2840  adj.P.Val=0.8520  N=6 | 0.9362  P=0.0460  adj.P.Val=0.0552  N=4 | 0.1377  P=0.7431  adj.P.Val=0.8480  N=5 | 0.7056  P=0.1759  adj.P.Val=0.5550  N=3 | -0.2514  P=0.5503  adj.P.Val=0.7619  N=5 | 0.8706  P=0.031  adj.P.Val=0.0857  N=6 | -0.8745  P=0.1598  adj.P.Val=0.2301  N=2 |
| *MYOM2* | -1.070  P=3.05E-04  adj.P.Val=0.011  N=5 | 0.7915  P=0.010  adj.P.Val=0.013  N=4 | -0.1009  P=0.7092  adj.P.Val=0.8480  N=5 | 0.4905  P=0.2158  adj.P.Val=0.5550  N=2 | -0.1561  P=0.6367  adj.P.Val=0.8490  N=3 | 0.1882  P=0.4590  adj.P.Val=0.6120  N=6 | 0.9444  P=0.0203  adj.P.Val=0.0349  N=2 |
| *TTN* | 0.7134  P=0.064  adj.P.Val=0.4606  N=2 | 0.3850  P=0.1773  adj.P.Val=0.1822  N=4 | -0.4173  P=0.1133  adj.P.Val=0.2253  N=5 | -0.1955  P=0.6030  adj.P.Val=0.8103  N=2 | 1.131  P=9.88E-04  adj.P.Val=0.0355  N=3 | 0.2613  P=0.2849  adj.P.Val=0.4854  N=6 | -0.8313  P=0.0323  adj.P.Val=0.0529  N=2 |
| *TNNT1* | -0.5042  P=0.2640  adj.P.Val=0.8520  N=6 | 1.3264  P=0.0134  adj.P.Val=0.0166  N=4 | 0.5948  P=0.2502  adj.P.Val=0.4290  N=4 | 1.4525  P=0.0361  adj.P.Val=0.1623  N=2 | 0.4188  P=0.3780  adj.P.Val=0.6473  N=5 | 1.3925  P=0.00348  adj.P.Val=0.0139  N=6 | -1.7008  P=0.0152  adj.P.Val=0.0274  N=2 |
| *TNNT3* | 0.1668  P=0.7814  adj.P.Val=0.9621  N=6 | -0.5030  P=0.4667  adj.P.Val=0.4667  N=4 | -0.2161  P=0.7538  adj.P.Val=0.8480  N=4 | -5.1010  P=1.97E-06  adj.P.Val=2.36E-05  N=2 | -0.6090  P=0.3424  adj.P.Val=0.6164  N=5 | -3.4843  P=1.29E-06  adj.P.Val=1.55E-05  N=6 | 0.8509  P=0.3483  adj.P.Val=0.4478  N=2 |

Moderated t-statistics was calculated using the limma package.^31^ Obtained P-values were adjusted for multiple testing using Benjamini-Hochberg method to control the false discovery rate

**Supplementary Table 9:** Mean log_2_ fold change in expression of transcripts encoding enzymes involved in epigenetic modifications, in muscles of patients with CM and foetal muscles

|  | **Patient category and mutated gene**  **(mean log2 fold change in expression, P values, adj. P.Values and N samples )** | | | | | |  |
| --- | --- | --- | --- | --- | --- | --- | --- |
| **Gene Target** | Rhabdomyolysis  (AD *RYR1*) | XL-MTM  (*MTM1*) | MmD (*SEPN1/SELENON*) | CCD  (AD *RYR1*) | MmD  (AR *RYR1*) | Nemaline  (AD *KBTBD13*) | Fetus |
| *DNMT1* | -0.1812  P=0.7915  adj.P.Val=0.9621  N=5 | 5.7941  P=1.81E-12  adj.P.Val=6.50E-11  N=6 | 2.7521  P= 1.10E-05  adj.P.Val=6.61E-05  N=8 | 1.073  P=0.2122  adj.P.Val=0.5549  N=3 | 0.6331  P= 0.4595  adj.P.Val=0.6893  N=3 | 0.8030  P=0.2104  adj.P.Val=0.4734  N=6 | 0.2146  P=0.8344  adj.P.Val=0.8582  N=2 |
| *TRDMT1* | -0.2927  P=0.6843  adj.P.Val=0.9621  N=5 | 5.9157  P=3.86E-12  adj.P.Val=6.96E-11  N=6 | 1.7933  P= 0.0019  adj.P.Val=0.0063  N=10 | 0.3367  P=0.7070  adj.P.Val=0.8103  N=3 | -0.2088  P= 0.8156  adj.P.Val=0.9022  N=3 | -0.4445  P=0.5069  adj.P.Val=0.6437  N=6 | 0.076  P=0.9440  adj.P.Val=0.9440  N=2 |
| *DNMT3A* | 0.3609  P=0.2425  adj.P.Val=0.852  N=6 | 3.0996  P=4.12E-11  adj.P.Val=3.71E-10  N=4 | -0.0645  P= 0.8440  adj.P.Val=0.9207  N=5 | 1.161  P=0.0508  adj.P.Val=0.030  N=3 | 0.4328  P=0.1625  adj.P.Val=0.4179  N=6 | 0.5100  P=0.1013  adj.P.Val=0.2604  N=6 | 6.5372  P=2.02E-17  adj.P.Val=7.28E-16  N=2 |
| *HDAC1* | -0.1788  P=0.6504  adj.P.Val=0.9621  N=7 | 2.6131  P=1.73E-06  adj.P.Val=4.16E-06  N=4 | -1.7858  P= 8.65E-07  adj.P.Val=7.78E-06  N=12 | 0.2340  P=0.6757  adj.P.Val=0.8103  N=3 | 0.1649  P= 0.6937  adj.P.Val=0.8649  N=6 | -0.4879  P=0.2462  adj.P.Val=0.4855  N=6 | 3.5842  P=2.44E-08  adj.P.Val=1.26E-07  N=3 |
| *HDAC3* | -0.0210  P=0.9441  adj.P.Val=0.9621  N=6 | 2.1458  P=9.62E-08  adj.P.Val=2.66E-07  N=4 | -1.9442  1.90E-11  adj.P.Val=6.83E-10  N=12 | 0.2772  P=0.4882  adj.P.Val=0.7990  N=3 | 0.1167  P= 0.6967  adj.P.Val=0.8649  N=6 | -0.0282  P=0.9250  adj.P.Val=0.9515  N=6 | 2.6256  P=1.45E-08  adj.P.Val=8.71E-0.8  N=3 |
| *HDAC4* | -0.0562  P=0.9233  adj.P.Val=0.9621  N=7 | 3.9564  P=2.09E-08  adj.P.Val=6.83E-08  N=6 | 0.3554  P= 0.4593  adj.P.Val=0.6124  N=12 | 0.6071  P=0.4676  adj.P.Val=0.7990  N=3 | 1.5884  P= 0.0081  adj.P.Val=0.0635  N=7 | -0.5050  P=0.4168  adj.P.Val=0.5847  N=6 | 0.5003  P=0.6182  adj.P.Val=0.6744  N=2 |
| *HDAC5* | 0.0404  P=0.9364  adj.P.Val=0.9621  N=7 | 2.1627  P=0.00118  adj.P.Val=0.00163  N=4 | 2.3322  P= 4.66E-07  adj.P.Val=5.59E-06  N=12 | 0.4528  P=0.5327  adj.P.Val=0.7990  N=3 | 1.2467  P= 0.0163  adj.P.Val=0.0734  N=7 | 0.5958  P=0.2714  adj.P.Val=0.4853  N=6 | 1.9283  P=0.0096  adj.P.Val =0.0192  N=3 |
| *HDAC9* | -0.0952  P=0.8168  adj.P.Val=0.9621  N=4 | 3.1408  P=1.18E-09  adj.P.Val=6.05E-09  N=4 | 1.5168  P= 1.93E-04  adj.P.Val=9.92E-04  N=5 | 0.3232  P=0.4880  adj.P.Val=0.7990  N=3 | -0.5838  P= 0.2131  adj.P.Val=0.5116  N=3 | 0.3661  P=0.2966  adj.P.Val=0.4853  N=6 | 3.9812  P=6.33E -09  adj.P.Val=4.56E-08  N=2 |

Moderated t-statistics was calculated using the limma package.^31^ Obtained P-values were adjusted for multiple testing using Benjamini-Hochberg method to control the false discovery rate.

**Supplementary Table 10:** Mean log_2_ fold change in expression of transcripts encoding transcription factors and splicing regulators, in muscles of patients with CM and foetal muscle

|  | **Patient category and mutated gene**  **(mean log2 fold change in expression, P values, adj. P.Values and N samples )** | | | | | |  |
| --- | --- | --- | --- | --- | --- | --- | --- |
| **Gene Target** | Rhabdomyolysis  (AD *RYR1*) | XL-MTM  (*MTM1*) | MmD (*SEPN1/SELENON*) | CCD  AD *RYR1* | MmD  (AR*RYR1*) | Nemaline  (AD *KBTBD13*) | Fetus |
| *NFATC2* | -0.6133  P= 0.0450  adj.P.Val=0.4048  N=6 | 1.4471  P= 1.42E-04  adj.P.Val=2.22E-04  N=4 | -0.0108  P= 0.9732  adj.P.Val=0.9732  N=5 | 0.1154  P= 0.7689  adj.P.Val=0.8388  N=3 | -0.2063  P= 0.4912  adj.P.Val=0.7073  N=6 | 0.7376  P= 0.0170  adj.P.Val=0.0556  N=6 | 2.8091  P= 3.08E-07  adj.P.Val=1.01E-06  N=2 |
| *NFATC3* | 0.0163  P= 0.9530  adj.P.Val=0.9621  N=6 | 1.8307  P= 1.35E-06  adj.P.Val=3.48E-06  N=4 | 0.5026  P= 0.1189  adj.P.Val=0.2253  N=4 | -0.2664  P= 0.5216  adj.P.Val=0.7990  N=2 | 0.0641  P= 0.8270  adj.P.Val=0.9022  N=5 | 0.0983  P=0.7222  adj.P.Val=0.8387  N=6 | 2.8941  P=3.63E-08  adj.P.Val=1.45E-07  N=2 |
| *MEF2A* | 0.3918  P= 0.1771  adj.P.Val=0.7970  N=6 | 2.2995  P= 3.90E-08  adj.P.Val=1.17E-07  N=4 | 0.6824  P= 0.0437  adj.P.Val=0.1048  N=4 | 0.2031  P= 0.6368  adj.P.Val=0.8103  N=2 | 0.3228  P= 0.2919  adj.P.Val =0.5837  N=5 | 0.3301  P= 0.2813  adj.P.Val=0.4854  N=5 | 2.5887  P=7.16E-07  adj.P.Val=2.15E-06  N=2 |
| *MEF2C* | -0.3798  P= 0.1154  adj.P.Val=0.5934  N=7 | 2.2663  P= 1.87E-09  adj.P.Val=8.43E-09  N=4 | -0.1531  P= 0.5671  adj.P.Val=0.7291  N=5 | 0.0423  P= 0.9130  adj.P.Val=0.9160  N=2 | -0.6122  P= 0.0664  adj.P.Val=0.2173  N=3 | 0.9062  P= 0.0015  adj.P.Val=0.0069  N=5 | 1.8178  P= 3.18E-05  adj.P.Val=7.62E-05  N=2 |
| *MEF2D* | 0.0609  P= 0.7465  adj.P.Val=0.9621  N=6 | 1.1753  P= 4.10E-06  adj.P.Val=8.82E-06  N=4 | 0.1859  P= 0.3938  adj.P.Val=0.5670  N=4 | -0.0374  P=0.8946  adj.P.Val=0.9160  N=2 | 0.1696  P=0.3983  adj.P.Val=0.6474  N=5 | 0.0596  P= 0.7658  adj.P.Val=0.8615  N=5 | 1.5050  P= 5.87E-06  adj.P.Val=1.51E-05  N=2 |
| *MBNL1* | -0.6252  P= 0.0062  adj.P.Val=0.0742  N=6 | 1.1943  P= 2.55E-05  adj.P.Val=4.84E-05  N=4 | -0.1806  P= 0.4417  adj.P.Val=0.6116  N=5 | -0.1029  P= 0.7203  adj.P.Val=0.8103  N=3 | -0.5726  P= 0.0116  adj.P.Val=0.0635  N=6 | 0.0014  P= 0.9951  adj.P.Val=0.9951  N=6 | 0.4509  P=0.1922  adj.P.Val=0.2662  N=2 |

Moderated t-statistics was calculated using the limma package.^31^ Obtained P-values were adjusted for multiple testing using Benjamini-Hochberg method to control the false discovery rate.

**Supplementary Table 11:** Mean log_2_ fold change in expression of miRNAs targeting transcripts encoding proteins involved in ECC, muscle, epigenetic and signalling related transcripts, in muscles of patients with CM and foetal muscles

|  | **Patient category and mutated gene**  **(mean log2 fold change in expression, P values, adj. P.Values and N samples)** | | | | | |  |
| --- | --- | --- | --- | --- | --- | --- | --- |
| **hsa-miR** | Rhabdomyolysis  (AD *RYR1*) | XL-MTM  (*MTM1*) | MmD (*SEPN1/SELENON*) | CCD  (AD *RYR1*) | MmD  (AR *RYR1*) | Nemaline  (AD *KBTBD13*) | Fetus |
| miR-1-3p | 1.1196  P= 0.2971  adj.P.Val=0.9746  N=3 | -3.4437  P= 4.10E-05  adj.P.Val=9.03E-05  N=6 | -1.7386  P= 0.0058  adj.P.Val=0.0159  N=12 | -1.1844  P= 0.1396  adj.P.Val=0.2558  N=6 | -2.0610  P= 3.25E-04  adj.P.Val=0.0033  N=17 | -1.8242  P= 0.0020  adj.P.Val=0.0108  N=15 | -3.1830  P= 0.0152  adj.P.Val=0.0217  N=2 |
| miR-95-3p | -0.1354  P= 0.7508  adj.P.Val=0.9746  N=4 | -2.4225  P= 2.38E-06  adj.P.Val=6.53E-06  N=4 | -0.1499  P= 0.7251  adj.P.Val=0.8863  N=4 | -1.8084  P= 5.41E-04  adj.P.Val=0.006  N=3 | -0.6337  P= 0.1437  adj.P.Val=0.2258  N=4 | -1.4976  P= 2.97E-04  adj.P.Val=0.0033  N=6 | -5.972  P= 3.43E-12  adj.P.Val=1.71E-11  N=2 |
| miR-133a-3p | -0.5399  P= 0.7166  adj.P.Val=0.9746  N=3 | -5.6651  P= 1.82E-06  adj.P.Val=6.53E-06  N=6 | -2.1793  P= 0.0107  adj.P.Val=0.0236  N=13 | 0.8642  P= 0.4310  adj.P.Val=0.4310  N=6 | -0.9916  P= 0.2014  adj.P.Val=0.2770  N=17 | -2.7302  P= 0.00354  adj.P.Val=0.0130  N=10 | -3.9739  P= 0.0257  adj.P.Val=0.0321  N=2 |
| miR-133b-3p | -0.0105  P= 0.9795  adj.P.Val=0.9795  N=4 | -1.4227  P= 0.0015  adj.P.Val=0.0024  N=4 | 0.0967  P= 0.8134  adj.P.Val=0.8948  N=4 | -0.4823  P= 0.2932  adj.P.Val=0.4016  N=3 | -0.2668  P=0.5164  adj.P.Val=0.5347  N=4 | -0.5645  P= 0.1751  adj.P.Val=0.3210  N=4 | -1.8162  P= 0.0018  adj.P.Val=0.0045  N=2 |
| miR-206-3p | 0.7024  P= 0.5176  adj.P.Val=0.9746  N=3 | -0.2094  P=0.7968  adj.P.Val=0.8765  N=6 | 0.3684  P= 0.5528  adj.P.Val=0.8687  N=13 | 2.0906  P= 0.0120  adj.P.Val=0.0657  N=6 | 1.3142  P= 0.0243  adj.P.Val=0.0669  N=17 | 0.8652  P= 0.2382  adj.P.Val=0.3743  N=8 | 1.9182  P= 0.1431  adj.P.Val=0.1590  N=2 |
| miR-486-3p | 1.2259  P= 0.1067  adj.P.Val=0.9746  N=3 | -2.4225  P= 5.41E-04  adj.P.Val=9.92E-04  N=6 | -1.3906  P= 0.0015  adj.P.Val=0.0054  N=13 | -1.1055  P= 0.0516  adj.P.Val=0.1418  N=6 | -0.6629  P= 0.0903  adj.P.Val=0.1656  N=17 | 0.3192  P= 0.6309  adj.P.Val=0.7711  N=4 | -5.1157  P= 2.98E-07  adj.P.Val=9.63E-07  N=2 |
| miR-22-3p | 0.2350  P=0.7974  adj.P.Val=0.9746  N=3 | -5.2781  P= 2.25E-11  adj.P.Val=1.24E-10  N=6 | -1.9335  P= 5.68E-04  adj.P.Val=0.0031  N=11 | -1.3982  P= 0.0424  adj.P.Val=0.1419  N=6 | -1.5840  P= 0.0013  adj.P.Val=0.0482  N=17 | -1.4180  P=0.0054  adj.P.Val=0.015  N=14 | -2.8404  P=0.0115  adj.P.Val=0.0192  N=2 |
| miR-124-3p | 0.1541  P=0.9034  adj.P.Val=0.9795  N=3 | 0.3566  P= 0.7122  adj.P.Val=0.8705  N=6 | -0.6953  P= 0.4097  adj.P.Val=0.7512  N=9 | -0.8783  P= 0.3651  adj.P.Val=0.4015  N=6 | -2.5724  P= 6.07E-04  adj.P.Val=0.0033  N=17 | -0.6325  P= 0.4702  adj.P.Val=0.6466  N=8 | Not detected |
| miR-193b-3p | 0.2669  P= 0.4887  adj.P.Val=0.9746  N=4 | -2.8152  P= 1.21E-13  adj.P.Val=1.34E-12  N=6 | -1.7420  P= 8.82E-10  adj.P.Val=9.79E-09  N=10 | -0.6395  P=0.0978  adj.P.Val=0.2152  N=3 | -0.5947  P= 0.0814  adj.P.Val=0.1656  N=4 | 0.0251  P= 0.9403  adj.P.Val=0.9403  N=4 | -4.4808  P= 1.90E-13  adj.P.Val=1.90E-12  N=2 |
| miR-221-3p | 0.3387  P= 0.6872  adj.P.Val=0.9746  N=3 | -0.0697  P=0.9146  adj.P.Val=0.9146  N=6 | 0.0207  P= 0.9746  adj.P.Val=0.9746  N=6 | -0.6267  P= 0.3381  adj.P.Val=0.4016  N=6 | -0.3186  P=0.5347  adj.P.Val=0.5347  N=14 | -0.2633  P=0.7263  adj.P.Val=0.7990  N=4 | 1.2877  P= 0.1992  adj.P.Val=0.1992  N=2 |
| miR-16-5p | -0.5783  P= 0.2843  adj.P.Val=0.9746  N=4 | -1.5004  P= 0.0081  adj.P.Val=0.011  N=4 | -0.2524  P= 0.6378  adj.P.Val=0.8770  N=4 | -0.8212  P= 0.1742  adj.P.Val=0.2738  N=3 | -0.3824  P= 0.4767  adj.P.Val=0.5347  N=4 | -1.1787  P= 0.0160  adj.P.Val=0.0135  N=6 | -2.240  P= 0.0029  adj.P.Val=0.0059  N=2 |

Moderated t-statistics was calculated using the limma package.^31^ Obtained P-values were adjusted for multiple testing using Benjamini-Hochberg method to control the false discovery rate.

**Supplementary References**

1. MacLennan DH, Duff C, Zorzato F, *et al.* Ryanodine receptor gene is a candidate for predisposition to malignant hyperthermia. *Nature*. 1990; 343: 559–561.
2. Tanabe T, Takeshima H, Mikami A, *et al*. Primary structure of the receptor for calcium channel blockers from skeletal muscle. *Nature*. 1987; 328: 313–318.
3. Nelson BR, Wu F, Liu Y, *et al*. Skeletal muscle-specific T-tubule protein STAC3 mediates voltage-induced Ca^2+^ release and contractility. *Proc Natl Acad Sci USA.* 2013; 110: 11881–1186.
4. Polster A, Perni S, Bichraoui H, Beam KG. Stac adaptor proteins regulate trafficking and function of muscle and neuronal L-type Ca ^2+^ channels. P*roc Natl Acad Sci USA.* 2015; 112: 602–606.
5. MacLennan DH. Ca^2+^ signalling and muscle disease. *Eur J Biochem*. 2000; 267: 5291–5297.
6. Carafoli E. Calcium pump of the plasma membrane. *Physiol Rev*. 1991; 71:129-153.
7. Qiu R, Lewis RS. Structural features of STIM and Orai underlying store-operated calcium entry. *Curr Opin Cell Biol*. 2019; 57: 90–98.
8. Feng W, Tu J, Yang T, *et al*. Homer Regulates Gain of Ryanodine Receptor Type 1 Channel Complex. *J Biol Chem*. 2002; 277: 44722–44730.
9. Shin DM, Dehoff M, Luo X, *et al*. Homer 2 tunes G protein–coupled receptors stimulus intensity by regulating RGS proteins and PLCβ GAP activities. *J Cell Biol.* 2003; 162: 293–303.
10. Michikawa T, Miyawaki A, Furuichi T, Mikoshiba K. Inositol 1,4,5-Trisphosphate Receptors and Calcium Signaling. Crit Rev Neurobiol. 1996; 10: 39–55.
11. Schiaffino S, Reggiani C. Fiber Types in Mammalian Skeletal Muscles. *Physiol Rev.* 2011; 91: 1447–1531.
12. Obermann WMJ. Molecular structure of the sarcomeric M band: mapping of titin and myosin binding domains in myomesin and the identification of a potential regulatory phosphorylation site in myomesin. *EMBO J*. 1997; 16: 211–220.
13. Lyko F. The DNA methyltransferase family: a versatile toolkit for epigenetic regulation. *Nat Rev Genet.*. 2018; 19: 81–92.
14. Haberland M, Montgomery RL, Olson EN. The many roles of histone deacetylases in development and physiology: implications for disease and therapy. *Nat Rev Genet*. 2009; 10: 32–42.
15. Chin ER, Olson EN, Richardson JA, *et al*. Calcineurin-dependent transcriptional pathway controls skeletal muscle fiber type. *Genes Dev*. 1998; 12: 2499-2509.
16. McKinsey TA, Zhang CL, Lu J, Olson EN. Signal dependent nuclear export of a histone deacetylase regulates muscle differentiation. *Nature*. 2000; 408:106-111.
17. Lu J, McKinsey TA, Nicol RL, Olson EN. Signal-dependent activation of the MEF2 transcription factor by dissociation from histone deacetylase. *Proc Natl Acad Sci USA*. 2000; 97: 4070-4075.
18. Wang ET, Cody NAL, Jog S, Biancolella M, *et al*. Transcriptome-wide regulation of pre-mRNA splicing and mRNA localization by muscleblind proteins. *Cell* 2012; 150: 710-724.
19. Witting N, Laforêt P, Voermans NC, *et al*. Phenotype and genotype of muscle ryanodine receptor rhabdomyolysis-myalgia syndrome. *Acta Neurol Scand*. 2018; 137:452-461
20. Richter M, Schleithoff L, Deufel T, Lehmann-Horn F, Herrmann-Frank A. Functional characterization of a distinct ryanodine receptor mutation in human malignant hyperthermia -susceptible muscle. *J Biol Chem*. 1997; 272: 5256-5260.
21. Manning BM, Quane KA, Ording H, Urwyler A, *et al.* Identification of novel mutations in the ryanodine receptor gene (RYR1) in malignant hyperthermia: genotype-phenotype correlation. *Am J Hum Genet* 1998; 62: 599-609.
22. Shephered S, Ellis F, Halsall J, Hopkins P, Robinson R. RYR1 mutations in UK central core disease patients: more than just C-terminal transmembrane region of the RYR1 gene. *J Med Genet*. 2004; 41 (3) e33.
23. Dlamini N, Voermans NC, Lillis S, *et al*. Mutations in RYR1 are a common cause of exertional myalgia and rhabdomyolysis. *Neuromuscul Disord.* 2013; 23:540-548,
24. Keting KE, Giblin L, Lynch PJ, *et al*. Detection of a novel mutation in the ryanodine receptor gene in an Irish malignant hyperthermia pedigree: correlation of the IVCT response with the affected and unaffected haplotypes. *J Med Genet.* 1997; 34:291-296.
25. Rokach O, Sekulic-Jablanovic M, Voermans N, *et a*l. Epigenetic changes as a common trigger of muscle weakness in congenital myopathies. *Hum Mol Genet*. 2015; 24: 4636–4647.
26. Wilmshurst JM, Lillis S, Zhou H, *et al*. RYR1 mutations are a common cause of congenital myopathies with central nuclei. *Ann Neurol*. 2010; 68: 717-726.
27. Gillard EF, Otsu K, Fujii J, *et al*. A substitution of cysteine for arginine 614 in the ryanodine receptor is potentially causative of human malignant hyperthermia. *Genomics* 1991: 11:751-755.
28. Sato I, Wu S, Ibarra MC, *et al*. Congenital neuromuscular diseae with uniform type 1 fiber and RYR1 mutation. *Neurology* 2008; 70: 114-122.
29. Bachmann C, Noreen F, Voermans NC, Schär PL, Vissing J, Fock JM*, et al*. Aberrant regulation of epigenetic modifiers contributes to the pathogenesis in patients with selenoprotein N ‐ related myopathies. *Hum Mutat.* 2019; 40: 962–974.
30. Boumann K, Küsters B, De Winter JM, *et al*. NEM6, KBTBD13-related congenitla myopathy: myopathological analysis in 18 Dutch patients reveals ring rods fibers, cores, nuclear clumps, and granulo-filamentous protein material. *J Neuropathol Exp Neurol.* 2021; 22: 366-376.
31. Ritchie ME, Phipson B, Wu D, Hu Y, Law CW, Shi W, Smyth GK. Limma powers differential expression analyses for RNA-sequencing and microarray studies. Nucleic Acids Research 2015; 43(7), e47. doi: [10.1093/nar/gkv007](https://doi.org/10.1093/nar/gkv007).
